# Supplementary material for: Demography and perturbation analyses of the coffee berry borer Hypothenemus hampei (Coleoptera: Curculionidae): Implications for management
Source: PLoS One. 2021 Dec 14;16(12):e0260499. doi: 10.1371/journal.pone.0260499 (PMC8670699; doi:10.1371/journal.pone.0260499)
Supplement: S1 Table — (PDF) [file pone.0260499.s001.pdf]

Supporting material

S1 Table. Projection matrix for populations of the coffee berry borer (CBB, *Hypothenemus hampei*) reared in lab on the artificial diet CENIBROCA at temperature of 25 ± 1°C, relative humidity 80 – 96%

Egg	Larvae	Pupae	Juvenil	Adult
0.09	0	0	0	11.39
0.90	0.1	0	0	0
0	0.85	0.18	0	0
0	0	0.78	0.21	0
0	0	0	0.79	0.91
