# Supplementary material for: Demography and perturbation analyses of the coffee berry borer Hypothenemus hampei (Coleoptera: Curculionidae): Implications for management
Source: PLoS One. 2021 Dec 14;16(12):e0260499. doi: 10.1371/journal.pone.0260499 (PMC8670699; doi:10.1371/journal.pone.0260499)
Supplement: S2 Table — (PDF) [file pone.0260499.s002.pdf]

Table S2. Projection matrix for populations of the coffee berry borer (CBB, *Hypothenemus hampei*) artificial infestations in a shade coffee plot planted with *Coffeea arabica* cv. Limaní, mean temperature 21.8 ± 3°C, relative humidity ranged from 42.5 to 100%.

Egg	Larvae	Pupae	Juvenil	Adult
0	0	0	0	10.43
0.89	0	0	0	0
0	0.57	0	0	0
0	0	0.39	0	0
0	0	0	0.75	0.80
