# Supplementary material for: Demography and perturbation analyses of the coffee berry borer Hypothenemus hampei (Coleoptera: Curculionidae): Implications for management
Source: PLoS One. 2021 Dec 14;16(12):e0260499. doi: 10.1371/journal.pone.0260499 (PMC8670699; doi:10.1371/journal.pone.0260499)
Supplement: Attachment — Submitted filename: PONE-D-21-18910_reviewer_ok.pdf [file pone.0260499.s004.pdf]

PLOS ONE

Demography and perturbation analyses of the coffee berry borer <i>Hypothenemus hampei</i> (Coleoptera: Curculionidae): implications for management

--Manuscript Draft--

**Manuscript Number:**	PONE-D-21-18910
**Article Type:**	Research Article
**Full Title:**	Demography and perturbation analyses of the coffee berry borer <i>Hypothenemus hampei</i> (Coleoptera: Curculionidae): implications for management
**Short Title:**	Demography and perturbation analyses of the CBB <i>Hypothenemus hampei</i>
**Corresponding Author:**	Yobana Andrea Mariño, PhD.<br>University of Puerto Rico Rio Piedras: Universidad de Puerto Rico Recinto de Rio Piedras<br>San Juan, PR PUERTO RICO
**Keywords:**	<i>Hypothenemus hampei</i>; sensitivity; life table; perturbation analysis; pest management
**Abstract:**	The coffee berry borer (CBB) <i> Hypothenemus hampei</i> Ferrari is the most serious coffee pest worldwide. Management of the CBB is extremely difficult because its entire life cycle occurs well-protected inside the fruit. Knowing which specific life stages contribute most to population growth would help understand its population dynamics and help to improve management programs for the CBB. Two staged-classified matrices or Leslie matrices were constructed for CBB populations reared in the lab on artificial diets and CBBs from artificial infestations in the field. Matrices were used to determine demographic parameters, to conduct elasticity analyses, and to perform prospective perturbation analysis. Elasticity analyses indicated that the transition from larva to pupa, transition from pupa to juvenile and survival of adult females were the three vital rates with largest contribution to λ (population growth rate) for both populations; these vital rates can be important targets for CBB management. Perturbation analyses indicated that an effective management for the CBB should not focus on only one vital rate; perturbations of >90% for each developmental stage are necessary to reduce λ to <1. However, when the three vital rates with highest sensitivity are impacted at the same time, the percentage of perturbation is reduced to 25% for each transition. Management programs for CBB should be focused on the use of biological and cultural measures that are known to affect these three important targets. Natural enemies like ants and parasitoids play an important role in reducing the survival of larvae and pupae; the fungus <i> Beauveria bassiana</i> (Balsamo) Vulleimen, parasitoids and cultural practices like sanitation after the harvest are important tools to reduce the survival of CBB females.
**Order of Authors:**	Yobana Andrea Mariño, PhD.
	Paul Bayman
	Alberto Sabat
**Additional Information:**	
**Question**	**Response**
**Financial Disclosure**<br><br>Enter a financial disclosure statement that describes the sources of funding for the work included in this submission. Review the submission guidelines for detailed requirements. View published research articles from *PLOS ONE* for specific examples.	PB, USDA ARS Specific Cooperative Agreements 58-1245-4-082 and 58-1245-4-083.<br><br>The funders had no role in study design, data collection and analysis, decision to publish, or preparation of the manuscript.

Powered by Editorial Manager® and ProduXion Manager® from Aries Systems Corporation

This statement is required for submission and **will appear in the published article** if the submission is accepted. Please make sure it is accurate.<br><br>**Unfunded studies**<br>Enter: *The author(s) received no specific funding for this work.*<br><br>**Funded studies**<br>Enter a statement with the following details:<br>• Initials of the authors who received each award<br>• Grant numbers awarded to each author<br>• The full name of each funder<br>• URL of each funder website<br>• Did the sponsors or funders play any role in the study design, data collection and analysis, decision to publish, or preparation of the manuscript?<br>• **NO** - Include this sentence at the end of your statement: *The funders had no role in study design, data collection and analysis, decision to publish, or preparation of the manuscript.*<br>• **YES** - Specify the role(s) played.<br><br>* typeset	
**Competing Interests**<br><br>Use the instructions below to enter a competing interest statement for this submission. On behalf of all authors, disclose any competing interests that could be perceived to bias this work—acknowledging all financial support and any other relevant financial or non-financial competing interests.<br><br>This statement is required for submission and **will appear in the published article** if the submission is accepted. Please make sure it is accurate and that any funding sources listed in your Funding Information later in the submission form are also declared in your Financial Disclosure statement.<br><br>View published research articles from *PLOS ONE* for specific examples.	The authors have declared that no competing interests exist.

Powered by Editorial Manager® and ProduXion Manager® from Aries Systems Corporation

**NO authors have competing interests**<br><br>Enter: *The authors have declared that no competing interests exist.*<br><br>**Authors with competing interests**<br><br>Enter competing interest details beginning with this statement:<br><br>*I have read the journal's policy and the authors of this manuscript have the following competing interests: [insert competing interests here]*<br><br>* typeset	
**Ethics Statement**<br><br>Enter an ethics statement for this submission. This statement is required if the study involved:<br><br>• Human participants<br>• Human specimens or tissue<br>• Vertebrate animals or cephalopods<br>• Vertebrate embryos or tissues<br>• Field research<br><br>Write "N/A" if the submission does not require an ethics statement.<br><br>General guidance is provided below. Consult the submission guidelines for detailed instructions. **Make sure that all information entered here is included in the Methods section of the manuscript.**	N/A

Powered by Editorial Manager® and ProduXion Manager® from Aries Systems Corporation

**Format for specific study types**<br><br>**Human Subject Research (involving human participants and/or tissue)**<br>• Give the name of the institutional review board or ethics committee that approved the study<br>• Include the approval number and/or a statement indicating approval of this research<br>• Indicate the form of consent obtained (written/oral) or the reason that consent was not obtained (e.g. the data were analyzed anonymously)<br><br>**Animal Research (involving vertebrate animals, embryos or tissues)**<br>• Provide the name of the Institutional Animal Care and Use Committee (IACUC) or other relevant ethics board that reviewed the study protocol, and indicate whether they approved this research or granted a formal waiver of ethical approval<br>• Include an approval number if one was obtained<br>• If the study involved *non-human primates*, add *additional details* about animal welfare and steps taken to ameliorate suffering<br>• If anesthesia, euthanasia, or any kind of animal sacrifice is part of the study, include briefly which substances and/or methods were applied<br><br>**Field Research**<br><br>Include the following details if this study involves the collection of plant, animal, or other materials from a natural setting:<br>• Field permit number<br>• Name of the institution or relevant body that granted permission	
**Data Availability**<br><br>Authors are required to make all data underlying the findings described fully available, without restriction, and from the time of publication. PLOS allows rare exceptions to address legal and ethical concerns. See the PLOS Data Policy and FAQ for detailed information.	Yes - all data are fully available without restriction

A Data Availability Statement describing where the data can be found is required at submission. Your answers to this question constitute the Data Availability Statement and **will be published in the article**, if accepted.<br><br>**Important:** Stating 'data available on request from the author' is not sufficient. If your data are only available upon request, select 'No' for the first question and explain your exceptional situation in the text box.<br><br>Do the authors confirm that all data underlying the findings described in their manuscript are fully available without restriction?	
**Describe where the data may be found in full sentences. If you are copying our sample text, replace any instances of XXX with the appropriate details.**<br><br>• If the data are **held or will be held in a public repository**, include URLs, accession numbers or DOIs. If this information will only be available after acceptance, indicate this by ticking the box below. For example: *All XXX files are available from the XXX database (accession number(s) XXX, XXX.).*<br>• If the data are all contained **within the manuscript and/or Supporting Information files**, enter the following: *All relevant data are within the manuscript and its Supporting Information files.*<br>• If neither of these applies but you are able to provide **details of access elsewhere**, with or without limitations, please do so. For example:<br><br>*Data cannot be shared publicly because of [XXX]. Data are available from the XXX Institutional Data Access / Ethics Committee (contact via XXX) for researchers who meet the criteria for access to confidential data.*<br><br>*The data underlying the results presented in the study are available from (include the name of the third party*	All relevant data are within the manuscript and its Supporting Information files

and contact information or URL).<br>• This text is appropriate if the data are owned by a third party and authors do not have permission to share the data.<br><br>* typeset	
Additional data availability information:	Tick here if your circumstances are not covered by the questions above and you need the journal's help to make your data available.

Powered by Editorial Manager® and ProduXion Manager® from Aries Systems Corporation

Manuscript Click here to access/download;Manuscript;renamed_38e1c.docx

Demography and perturbation analyses of the coffee berry borer *Hypothenemus hampei* (Coleoptera: Curculionidae): implications for management

Yobana A. **Mariño**[1*], Paul **Bayman**[1] and Alberto M. **Sabat**[1]

[1]University of Puerto Rico – Río Piedras, Department of Biology, PO Box 23360, San Juan, PR 00931-3360, USA

* Corresponding author: yandreamarinoc@gmail.com; yobana.marino@upr.edu (YAM)

All authors contributed equally to this work.

Abstract

The coffee berry borer (CBB) *Hypothenemus hampei* Ferrari is the most serious coffee pest worldwide. Management of the CBB is extremely difficult because its entire life cycle occurs well-protected inside the fruit. Knowing which specific life stages contribute most to population growth would help understand its population dynamics and help to improve management programs for the CBB. Two staged-classified matrices or Leslie matrices were constructed for CBB populations reared in the lab on artificial diets and CBBs from artificial infestations in the field. Matrices were used to determine demographic parameters, to conduct elasticity analyses, and to perform prospective perturbation analysis. Elasticity analyses indicated that the transition from larva to pupa, transition from pupa to juvenile and survival of adult females were the three vital rates with largest contribution to λ (population growth rate) for both populations; these vital rates can be important targets for CBB management. Perturbation analyses indicated that an effective management for the CBB should not focus on only one vital rate; perturbations of >90% for each developmental stage are necessary to reduce λ to <1. However, when the three vital rates with highest sensitivity are impacted at the same time, the percentage of perturbation is reduced to 25% for each transition. Management programs for CBB should be focused on the use of biological and cultural measures that are known to affect these three important targets. Natural enemies like ants and parasitoids play an important role in reducing the survival of larvae and pupae; the fungus *Beauveria bassiana* (Balsamo) Vulleimen, parasitoids and cultural practices like sanitation after the harvest are important tools to reduce the survival of CBB females.

Key Words: *Hypothenemus hampei;* sensitivity; life table; perturbation analysis; pest management

Introduction

Coffee is one of the most valuable commodities in the global market, and its exportation is a key source of income for many developing countries [1, 2]. Coffee is grown in more than 70 countries in the humid tropics [3]. In Latin America, coffee is produced from Mexico and the Caribbean to Brazil [4, 5].

The coffee berry borer (CBB) *Hypothenemus hampei* (Ferrari) is the most serious pest of coffee worldwide [3, 6, 7]. The CBB is endemic to central Africa but has been disseminated to most coffee-producing countries around the world [8, 9]. ~~CBB~~ females bore into the coffee fruit; when they reach the seed's endosperm, they build galleries and lay eggs. The entire life cycle is completed inside the fruit; only fertilized females abandon the fruit in search of new fruits to infest. Damage caused by the CBB is due to feeding activity of ~~adults~~ and their progeny in the coffee seeds, which reduce the quality and yield of the marketable product and induce the premature fall of green fruits. Additionally, holes made by the CBB can facilitate the entry of pathogens like bacteria and fungi, which cause internal rots [3, 6, 10].

Management of the CBB is extremely difficult, because the only appropriate moment to apply measures of control (chemical or biological) is when the adult females are out searching for new fruits to infest. One of the most important biological

insecticides used for management of CBB is the fungus *Beauveria bassiana* (Balsamo) Vulleimen, or Bb [11, 12]. In many countries, Bb is commonly applied using the commercial products Mycotrol® and Botanigard®.

Demographic perturbation models are an important tool to understand and manage populations, specifically those models that focus on sensitivity and elasticity analyses [13, 14]. These types of analyses can be used in two ways: to protect endangered species or to reduce populations of invasive pests and weeds [14]. However, most of these analyses have been focused on conservation of endangered populations and less on control of pests.

Sensitivity analysis estimates the effect of changes in vital rates (e.g., survival, growth, fecundity) on the population growth rate (λ). Elasticity is the proportional sensitivity and determines the proportional change in λ caused by a proportional change in a vital rate [13, 15, 16]. This type of analysis is called prospective analysis because it predicts the results of the vital rate perturbations before they happen. Prospective analysis can be used to identify potential management targets (vital rates with higher scores of sensitivities or elasticities), that can be modified to produce large changes in the value of λ [13].

Demographic studies or life table studies of the CBB have been done under both laboratory and field conditions [17-21]. These studies estimated important demographic parameters including: population growth rate (λ), net reproductive rate (R_0), intrinsic rate of natural increase (r_m) and mean generation time (T). Some of these studies showed that demographic parameters are affected by temperature, coffee variety and fruit age

[18-20]. However, none of these prior studies used demographic data to conduct perturbation analysis, specifically prospective analysis. Knowledge about population dynamics and sensitivity of vital rates with potential to be perturbed is useful for management decisions.

The goal of the present study was to perform artificial infestations of the CBB under field conditions and to rear the CBB under lab conditions, in order to estimate demographic transitions in its life cycle and to conduct perturbation analysis. The specific objectives were: (i) To compare demographic parameters of populations raised in the lab on an artificial diet (Cenibroca) with those from artificial infestations in the field. (ii) To identify the contribution of vital rates to differences in performance between populations raised in the lab vs. artificial infestation in field conditions. (iii) To compare the behavior in the field of females raised in the lab (artificial infestation) vs. those that naturally infest coffee fruits (natural infestation). (iv) To identify the vital rates most sensitive to perturbations, and (vi) to conduct prospective analysis with those vital rates than can be modified and used as important targets for the management of the CBB.

Materials and Methods

Insect rearing: Coffee berry borers ~~(CBBs)~~ were reared on the artificial diet Cenibroca [22] as previously described [21]. ~~CBBs~~ were maintained in a growth chamber (Model 818, Thermo Scientific, Dubuque, IA) set at 25ºC (±1ºC), 80 – 96% relative humidity (RH) and complete darkness. The colony was established in 2012. Founder females were obtained from infested fruits of *Coffea arabica* L. cv. Catuai from a farm in

Adjuntas, Puerto Rico (18°10’42” N, 66°44’36” W). The colony is maintained in the Biology Department of the University of Puerto Rico, Río Piedras.

Laboratory evaluations

For laboratory evaluations, 100 eggs from F_6 (6th generation) of the lab colony were transferred to tubes with fresh Cenibroca diet, one per tube. Eggs were evaluated daily for 50 days to determine the transition to the next developmental stage, survival in each stage and developmental time from egg to adult. To determine survival and fecundity of adult females, 100 females approximately ten days old were transferred individually to new tubes with diet and they were evaluated for 90 days. Monthly each female was removed from the diet and survival, oviposition and total population was determined. CBB individuals per tube (eggs, larvae, pre-pupae, pupae, juveniles) were counted, and original alive adult females were transferred again to new tubes with diet.

Populations of CBB evaluated:

CBBs raised in the lab: individuals reared in the lab on the artificial diet Cenibroca.

Artificial infestation: coffee fruits in field were infested by CBB females reared in the lab on the artificial diet Cenibroca.

Natural infestation: Coffee fruits infested by CBB females growing under natural conditions.

Artificial infestation in the field

Three infestations were made in August to September 2018; at this time of year most fruits are full-sized and are approximately 80 days old [23]. Infestations were carried out at the UPR Agricultural Experimental Station in Adjuntas, Puerto Rico (18° 10' 12" N, 66°47'43" W, altitude 434 m.a.s.l.) on *Coffea arabica* L. cv. Limaní growing under shade of *Gliricidia sepium* (mother-of-cocoa, Fabaceae: Faboideae).

For each infestation ten coffee trees were selected; in each tree five to ten branches with >30 green, full-sized fruits were tagged; fruits that showed perforations made by the CBB were manually removed. Approximately 90 branches were artificially infested. CBBs for artificial infestation were obtained from the colony maintained on the artificial diet Cenibroca described above.

On the day of infestation, CBB females were surface-disinfected with 1% sodium hypochlorite and 0.002% Tween 80, and rinsed twice with sterile distilled water (dH_2O) (1 min each). Then groups of fifty females were transferred to petri dishes (6 cm diameter) lined with a sterile moist filter paper and sealed with paraffin. Once in the field the CBBs were put into clear plastic bags (35 cm x 11 cm); bags were tied to the branches and removed two days later. Perforated fruits were marked with nail polish; a different color was used for each infestation. 50 infested berries were collected weekly from 7 to 56 days after infestation. Fruits were transported to the laboratory for dissection; all CBBs inside the fruits were counted (eggs, larvae I, larvae II, pre-pupae, pupae, juveniles, and adults) and oviposition, survival, presence and position of adult females was determined. Position of colonizing females was classified as follows: A) fruit is perforated but the colonizing female is not present; B) fruit is perforated but the female has not reached the endosperm; C) the female has reached the endosperm but there is no reproduction, and D) the female has made her gallery in the endosperm, and immature stages are found [24].

CBBs were noted as killed by the entomopathogenic fungus *B. bassiana* when a white mycelium typical of Bb was observed growing on dead colonizing females. Percent of internal fruit rot was evaluated following the scale described by Serrato-Diaz [10].

Natural infestation

Fifty fully feral field-infested fruits were collected weekly from August to December from the same plot where artificial infestations were performed. In the laboratory the same variables mentioned above were evaluated: total number of CBB individuals per fruit, survival, presence and position of colonizing females, number of CBB~~s~~ killed by the fungus *B. bassiana* and percentages of rotted fruits. Data from natural infestation was used to compare with the behavior of CBB~~s~~ from artificial infestation. However, data from natural infestations were not used to determine demographic parameters and sensitivity analysis, since the time of infestation was unknown.

No chemical or biological control for the pest was applied in this plot during the experiment to ensure that mortality was due to natural causes. Temperature and light intensity were continuously recorded using Onset Hobo data loggers (HOBO Pendant ® Temperature and light, 64K, Onset Computer®, Bourne, MA, USA). Three data loggers were located in the middle of the plot, hung on coffee plants 1 m above the ground. Daily precipitation and humidity were obtained from a HOBO RX 3000 weather station (Onset Computer®, Bourne, MA, USA), located in a plot nearby. Temperatures ranged

from 12.0 to 34.1°C with a mean temperature of 21.8°C; minimum relative humidity was 42.5 and maximum was 100%; and daily rainfall varied from 0 to 7.60 mm (Table 1).

Table 1. Environmental variables at the UPR Agricultural Experimental Station in Adjuntas, Puerto Rico. Mean ± S.E. and daily range temperature range. These environmental variables were recorded using HOBO Pendant ® dataloggers and HOBO RX300 weather station.

Temperature (°C)				Humidity (%)			Rain (mm)		
Min	Max	Mean	Daily range[a]	Min	Mean	Max	Min	Mean	Max
12.0	34.1	21.8 ± 3.02	14.1	42.5	89.5	100	0	0.02	7.60

Data analysis

For evaluation of both populations: CBBs raised in the lab and CBBs from artificial infestation in the field, the life cycle was divided into five developmental stages: egg, larva, pupa, juvenile and adult. Probability of surviving and remaining at the same life stage (P_i), probability of transition between stages, in which an individual survives and moves on to the next life stage (G_i), and fecundity of adult females (F_i) were calculated at the end of each evaluation, 50 days for lab and 56 days for field evaluations. Fecundity (F_i) was calculated as the proportion of females that oviposited multiplied by the mean number of eggs produced by each female. P_i probabilities were calculated only for CBBs reared in the lab; for field evaluations these probabilities could not be determined since the same individuals were not followed through time.

Sampling protocol and frequency of field evaluations did not allow determination of the exact duration of each developmental stage. Data on developmental times from

laboratory evaluations (Table 2) were used to calculate Gi or transitions between stages. Time of growth from egg to larva was estimated at 7 days; from larva to pupa 17 days; pupa to juvenile 7 days and juvenile to adult 4 days. To calculate G_1 (transition from egg to larva), the number of larvae counted at day 21 were divided by the number of eggs counted at day 14. Similarly, G_2 (transition from larva to pupa) was the proportion of pupae at day 42 divided by larvae in instar I counted at day 28. G_3 (transition from pupa to juvenile) was the proportion of juveniles counted at day 56 divided by the number of pupae at day 49. Finally, G_4 (transition from juveniles to adults) was determined as the proportion of adults at day 56 divided by the number of juveniles at day 56; the first juveniles were observed at day 56.

Staged-classified matrices, commonly known as Leslie matrices [25, 26], were constructed for both lab and field evaluations. The equation for a Leslie matrix is:

$$\mathrm{N}_{t+1} = \mathrm{A}\mathrm{N}_t$$

where N_t is a column vector representing the number of individuals in each developmental stage at time *t*: beginning of the evaluation. N_{t+1} is the number of individuals at the different stages at time *t+1:* end of the evaluation period, 50 days for lab evaluations and 56 days for field. **A** represents the *n* x *n* Leslie matrix or population projection matrix, given as follows:

$$\begin{bmatrix} P_1 & 0 & 0 & 0 & F_1 \\ G_1 & P_2 & 0 & 0 & 0 \\ 0 & G_2 & P_3 & 0 & 0 \\ 0 & 0 & G_3 & P_4 & 0 \\ 0 & 0 & 0 & G_4 & P_5 \end{bmatrix}$$

Entries in the first row of the matrix represent the fecundity rates. In the case of the CBB only the adult female can oviposit and contribute to population growth (F_1). The

main diagonal entries include the probabilities of surviving and staying in the same stage (P_i). The sub diagonal entries include the probability of surviving and advancing to the next stage (Gi). The zeros are transitions that do not occur in the life cycle of CBB.

Matrices were used to calculate the following demographic parameters: population growth rate (λ), intrinsic rate of natural increase (r_m), mean generation time (T), doubling time (TD), and sensitivity values. Sensitivity analysis estimates the effect of changes in vital rates (e.g., survival, growth, fecundity) on population growth rate (λ). Sensitivity matrix contains the sensitivities of λ_{max} to each element of the projection matrix $a_{i,j}$ [27], and is given by the equation:

$$S_{ij} = \frac{v_i w_i}{< w,v >} = \frac{\partial \lambda}{\partial a_{ij}}$$

where **w** and **v** are the right and left eigen vectors of the projection matrix **A** (the stable stage distribution and reproductive value, respectively) and ‹**w,v**› denotes the scalar product of **w** and **v** [28].

Perturbation analysis

Retroprospective analyses (LTRE or life table response experiment) measure the contribution of each vital rate in the variation of λ values for populations growing under different conditions or times [13]. Retroprospective analyses measure the contributions of past changes in vital rates and not the effects on future changes. LTRE were performed to determine which transitions or vital rates contributed to differences in values of λ between populations of CBBs raised in the lab vs. CBBs from artificial infestation in the field.

Prospective analyses (sensitivity and elasticity) predict changes in λ in response to modifications in vital rates that can be used to manage populations in the future. These changes in vital rates are independent of previous patterns of variability (environment or populations evaluated at different times) [13]. This type of analysis permits identification of potential vital rates that can be modified in order to decrease the value of λ, which is the objective for pests such as the CBB. Prospective analyses were done to estimate the change necessary in vital rates to decrease the value of λ to <1.0. To conduct prospective analysis, data from artificial infestation in the field were used for two reasons. First, the three vital rates with high sensitivities identified for both type of evaluations (field and lab) were the same (Table 4). Second, probabilities estimated for field evaluations reflect the true effects of biotic and abiotic variables on CBB populations.

Two types of analyses were conducted. First, all vital rates were modified including those with high sensitivity; in this case one vital rate was perturbed at a time holding the others constant. Second, two or three vital rates with higher sensitivities were lowered simultaneously to find the point at which the population growth rate fell below one.

Statistical analysis

The package popbio (version 2.4.3) was used to calculate all population parameters, generate elasticity matrices and perform perturbation analysis [29]. Generalized linear models (GLM) were used to compare female survival, presence,

positions inside the fruit and population per fruit between CBBs from artificial infestation vs. CBBs from natural infestation in the field. Models for proportion of alive, dead, missing and positions of colonizing females were fitted using a quasibinomial error distribution. A Poisson error distribution was used for CBB total population per fruit models. GLM models with a quasibinomial distribution of errors were used to compare proportion of internal fruit rot according to survival, abandon rate and positions of colonizing females; data from fruit rot was transformed to proportion. Function (predict) was used to calculate the posterior model probabilities. All analyses were done in R 3.5.3. [30].

Results

Comparisons between artificial infestations vs. natural infestations in the field

A total of 468 coffee fruits were successfully infested by CBB females (*H. hampei*) raised under lab conditions. After infestation, these CBBs behaved similarly to those in natural infestations. The proportion of missing, alive, and dead CBBs for both types of infestations is shown in Fig. 1. Neither the proportion of missing (χ^2_1 = 1.31, *p*=0.149) nor dead CBBs (χ^2_1 = 2.76, *p* =0.069) differed significantly between natural and artificial infestations. The only difference was in the proportion of alive CBBs, which was slightly higher for CBBs from artificial infestation (χ^2_1 = 3.43, *p* =0.009); the most notable difference was observed in August, when the proportion of live CBBs from natural infestation was 0.13 compared with 0.73 from artificial infestation (Fig. 1A). In August, a higher proportion of missing CBBs from natural (0.83) vs. artificial (0.21) infestations was also observed (Fig. 1C). These differences observed in August, at the

start of the experiment, may be a consequence of the short time that CBBs from artificial infestations were exposed to environmental or biological factors that kill them or stimulate their migration to other fruits; only a week had passed since the artificial infestation were done. In contrast, some of the fruits used for natural infestations may have been abandoned by CBBs before the experiment began.

Mortality from the fungus *B. bassiana* was not common. In only three fruits (<0.5%) the fungus was observed growing on a CBB cadaver. No significant difference was observed in internal rot between natural vs. artificial infestation ($\chi^2_1 = 0.51$, $p = 0.340$). Percentages of internal rots were significantly higher in fruits in which the colonizing CBB was dead or missing than in fruits where the CBB was alive ($\chi^2_2 = 21.9$, $p < 0.0001$); differences in percentages of fruit rot between dead vs. missing CBBs were not significant ($p = 0.126$). Estimated fruit rot was 3.7% for fruits with live CBBs; 7.9% for dead CBBs and 12.5% for fruits with missing CBBs.

Figure 1. Fate of coffee berry borers (CBB, *Hypothenemus hampei*) in artificial vs natural infestations in field after penetration of coffee fruits: proportion of alive, dead and missing CBBs. Artificial infestation: coffee fruits in field were infested by CBB females reared in lab on artificial diet Cenibroca. Natural infestation: coffee fruits infested by CBB in natural conditions. Sampling was done from August to December 2018 on a shade coffee plot of *Coffea arabica* cv. Limaní.

Positions of colonizing CBBs inside the fruit changed over time for both types of infestation (Fig. 2). Significant differences were found in positions C ($\chi^2_1 = 14.7$, $p < 0.0001$) and D ($\chi^2_1 = 20.3$, $p < 0.0001$) between artificial vs. natural infestations.

Proportions of CBBs in position C were higher for natural infestations (Fig. 2C). Conversely, proportions of CBBs in position D were higher for artificial infestation (Fig. 2D). This means that more CBBs reached the endosperm in naturally infested coffee, but they remained longer in this position (C) before starting to reproduce.

Figure 2. Progress of coffee berry borers (*Hypothenemus hampei*) in penetrating coffee fruits during the season. Artificial infestations: coffee fruits in field were infested by CBB females raised in lab on artificial diet Cenibroca. Natural infestation: coffee fruits infested by CBB in natural conditions. Sampling was done from August to December 2018 on a shade coffee plot of *Coffea arabica* cv. Limaní.

CBBs from artificial infestation produced more offspring per colonizing female than CBBs from natural infestation (Fig. 3). The estimated abundance of offspring in all developmental stages was 20.6 per fruit for artificial infestation vs. 4.5 for natural (χ^2_1 = 5473, p < 0.0001).

Estimated percentages of internal rots were significantly higher in fruits in which the colonizing female was in position C (χ^2_3 = 62.15, p < 0.0001). Estimated values were 7.2% for females in position A, 1.0% for position B, 20.1% for position C and 2.9% for position D.

Figure 3. Monthly changes in total number of individuals per fruit of the coffee berry borer (*Hypothenemus hampei*) in artificial vs. natural infestations. Means ± S.E. are shown. Artificial infestations: coffee fruits in the field were infested by CBB females raised in the lab on artificial

diet Cenibroca. Natural infestation: coffee fruits infested by CBB in natural conditions. Sampling was done from August to December 2018 on a shade coffee plot of *Coffea arabica* cv. Limani.

The abundance of individuals in each developmental stage for artificial infestation is shown in Fig 4. Eggs were found starting at day 7, and there were two peaks for eggs production at 14 and 35 days after infestation. Larvae had a pattern similar to eggs; they were present at all times and peaked at day 49. Pre-pupae appeared at day 35 and pupae at 42 days after infestation; both peaked at day 49. Juvenile females and males offspring appeared at same time, day 56. The first adult female offspring were observed on day 56 (the last sampling day). No adult males were observed. Developmental time from egg to adult under field conditions for a shade coffee plot with a mean temperature of 21.8 °C was approximately 49 days (Figure 4).

Figure 4. Number of CBBs (*Hypothenemus hampei*) per fruit in different developmental stages following artificial infestation: A, eggs and larvae; B, pre-pupae and pupae; C, juveniles; and D, adults. Coffee fruits were infested by CBB females raised in the lab on artificial diet Cenibroca. Means ± S.E. are shown. Sampling was done from August to December 2018 on a shade coffee plot of *Coffea arabica* cv. Limani.

Life cycle developmental times and life table parameters

Developmental time for CBBs reared in the lab on the artificial diet Cenibroca at 25 °C ranged from 32.9 to 35.9 days. Mean developmental times per stage were: 6 days from eggs to larvae, 15.2 days from larvae to pre-pupae, including both instars

(larva I and larva II), 2 days from pre-pupae to pupae, 6 days from pupae to juveniles and 4 days from juveniles to adults (Table 2).

Table 2. Development time in days for different stages of the coffee berry borer (*Hypothenemus hampei*) reared on artificial diet Cenibroca.

Stage	Media ± se	Minimum	Maximum	Confidence interval (95%)
Egg	6.0 ± 0.2	2.0	10.0	5.6 – 6.5
Larva	15.2 ± 0.7	8.0	20.0	13.7 – 16.7
Prepupa	1.9 ± 0.1	1.0	5.0	1.7 – 2.1
Pupa	6.3 ± 0.1	3.0	9.0	6.1 – 6.7
Juvenile	4.0 ± 0.1	6.0	3.0	3.8 – 4.3
Egg to adult	34.4 ± 0.7	21.0	40.0	32.9 – 35.9

The estimated population growth rate (λ) was over 1.0 for both populations; this means that in both cases CBB populations increased over its potential (Table 3). Mean generation time (T) and intrinsic rate of natural increase (r_m) were higher for populations reared in the lab on the artificial diet. The time of duplication (DT) was almost halved for populations raised in the lab. This reflects the lab conditions: an artificial diet with high nutritional content, constant temperature and relative humidity, unlike conditions in the field (Table 3).

Table 3. Life table parameters for populations of the coffee berry borer (*Hypothenemus hampei*) reared in the lab on artificial diet (Cenibroca) vs. in the field, artificial infestations made on *Coffee arabica* cv. Limani.

Cohort	Life Table Parameters			
	λ	T	r_m	DT
Lab [a]	1.74	41.5	0.058	11.8
Artificial infestations [b]	1.31	40.1	0.053	13.1

λ = population growth rate; T= mean generation time; r_m = intrinsic rate of natural increase: DT =doubling time (days)

[a] = individuals reared in the lab on the artificial diet Cenibroca (Temperature: 25 ± 1°C, RH 80 – 96%).

[b] = Individuals in the field on *Coffea arabica* cv. Limani following artificial infestations (Mean temperature 21.8 ± 3°C, RH 42.5 – 100%).

Perturbation analysis

Retroprospective analysis

According to LTRE analyses, the reduction in values for G_2 (transition from larva to pupa) and G_3 (transition from pupa to juvenile) for CBBs in the field made the largest contribution to the reduction in λ observed for these populations (Figure 5 and Table 3). Estimated transition or vital rates probabilities for populations raised in the lab and those in the field can be found in Table S1.

Figure 5. Life table response experiment (LTRE) analysis of the coffee berry borer (CBB, *Hypothenemus hampei*) reared in the lab vs. CBBs in the field (artificial infestations). The bars show the contribution of each life transition to the reduction in population growth rate (λ) when comparing between populations. G_i: probability of transition between stages, P_i: probability to survive and remain in the same stage, F_i: fertility of adult females. (1) Eggs, (2) Larvae, (3) Pupae, (4) Juveniles and (5) Adults.

Sensitivity analysis

Probabilities of transitions between stages (G_i) were higher than probabilities to survive and remain at the same stage (P_i). The three transitions with largest potential impact on population growth rate (λ) were: survival rates of adults (P_5: 0.324 for lab and 0.389 for field populations), transition from larva to pupa (G_2: 0.316 for lab and 0.352 for field) and transition from pupa to juvenile (G_3: 0.345 for lab and 0.515 for field) (Table 4).

Table 4. Sensitivities and elasticities for the coffee berry borer *Hypothenemus hampei* reared in the lab on the artificial diet (Cenibroca) and in the field, artificial infestations made on *Coffeea arabica* cv. Limani.

Vital rate	**Lab**		**Field**	
	**Sensitivities**	**Elasticities**	**Sensitivities**	**Elasticities**
G_1	0.299	0.154	0.225	0.049
G_2	**0.316**	0.154	**0.352**	0.049
G_3	**0.345**	0.154	**0.515**	0.049
G_4	0.240	0.154	0.267	0.049
P_1	0.163	0.008	-	-
P_2	0.164	0.009	-	-
P_3	0.172	0.017	-	-
P_4	0.175	0.021	-	-
P_5	**0.324**	0.169	**0.389**	0.703
F_1	0.023	0.154	0.019	0.049

G_1: probability of transition between stages, Pi: probability to survive and remain in the same stage, Fi: fertility of adult females. (1) Eggs, (2) Larvae, (3) Pupae, (4) Juveniles, (5) Adults. The sampling protocol for field evaluations did not allow us to determine the probability to survive and remain at the same stage (P_i) for some developmental stages

(eggs, larvae, pupae and juveniles). Transitions with largest potential impact on population growth rate (λ) are shown in **bold**.

Prospective perturbation analyses

The effects of proportional changes in vital transitions on the population growth rate (λ) for CBB populations in the field are shown in Figures 6 and 7 and Table 5. When only one parameter is perturbed at a time, including vital rates with the highest sensitivities (G_2, G_3 and P_5), no important changes in population growth rate were observed. When (G_i) (transition from egg to larva) was reduced by ≥70%, λ was < 1.0 (0.98), but for the remaining transitions the population growth rate was > 1.0 even when probabilities were reduced by ≥ 90% (Figure 7).

Figure 6. Coffee berry borer (*Hypothenemus hampei*) population growth rate (λ) as a function of: (A) Probability of transition between stages, (G1) eggs, (G2) larvae, (G3) pupae and (G4) juveniles; (B) survival of adult females and (C) fecundity of adult females, perturbing only one vital rate at a time. Data from field evaluations. λ value before the perturbations was 1.32.

Significant effects on population growth rate (λ) are possible when varying two or three transitions with higher sensitivities: that is, when G_2, G3 and P_5 are perturbed at the same time (Figure 7 and Table 5). When two transitions are perturbed at the same time reduction of 30% - 35% in both parameters is necessary to reduce the value of λ to < 1.0. For instance, a change of -35% for G_2 (0.60 to 0.25) and G_3 (0.40 to 0.05) produced a decrease of 30.3% in λ (1.32 to 0.92) (Figure 7A). Perturbing P_5 and G_3 at

the same time, only a 30% of reduction for each vital rate is necessary to decrease λ from 1.32 to 0.96; G_3 was the transition with highest sensitivity (Figure 7C).

Figure 7. Coffee berry borer (CBB, *Hypothenemus hampei*) population growth rate (λ) perturbing two transitions with higher elasticities at the same time: A) Perturbing G_2 and G_3; B) Perturbing G_2 and P_5; C) Perturbing G_3 and P_5. Data from field evaluations of artificial infestations on shade coffee plot *C. arabica* cv. Limani. λ value before the perturbations was 1.32.

When the three transitions are perturbed at the same time the disturbance percentage necessary to produce the same decrease in λ (1.32 to 0.96) is reduced to 25%. P_5 or adult survival should be lowered from 0.80 to 0.55; G_2 or transition from larva to pupa from 0.60 to 0.35, and G_3 or transition from pupa to juvenile from 0.40 to 0.15 (Table 5).

Table 5. Coffee berry borer (CBB, *Hypothenemus hampei*) population growth rate (λ) perturbing three transitions with higher elasticities at the same time. G_2: Transition from larva to pupa; G_3: Transition from pupa to juvenile, and P_5: Survival of adult females. Data from field evaluations of artificial infestations on *C. arabica* cv. Limaní. λ value before the perturbations was 1.32.

G_2	G_3	P_5	λ
0.60	0.40	0.80	1.33
0.55	0.35	0.75	1.27
0.50	0.30	0.70	1.20
0.45	0.25	0.65	1.13
0.40	0.20	0.60	1.05
**0.35**	**0.15**	**0.55**	**0.96**
0.30	0.10	0.50	0.86
0.25	0.05	0.45	0.74

Discussion

The principal aims of this study were to collect demographic data to perform retrospective and prospective perturbation analyses of coffee berry borer (CBB) populations. Prospective analyses are based on the determination of vital rates with highest sensitivities, which can be perturbed in order to decrease the value of population growth rate (λ) to < 1.0. Identification of vital rates with highest contributions to λ can be powerful tools to improve Integrated Pest Management (IPM) programs [14, 31].

Field and laboratory evaluations were conducted to determine demographic parameters for CBB populations. ~~Despite the importance of the CBB as a pest of coffee,~~ few studies have conducted field experiments to obtain demographic data [17, 20]. To the best of our knowledge, this is the first study to conduct field evaluations of the CBB to perform prospective perturbation analysis. Three vital rates were identified as important targets for CBB management: G_2 (transition from larva to pupa), G_3 (transition from pupa to juvenile) and P_5 (survival of adult females); these vital rates had the highest sensitivities, and their perturbation is biologically feasible (Table 4).

Perturbation analysis indicated that an efficient management for the CBB should not be focused on only one developmental stage. When one vital rate was perturbed at a time and the others remained constant, high values of change in each vital rate were necessary to reduce the value of λ to < 1.0. G_2 and G_3 had to be reduced more than 90% (Fig. 6A); for P_5, not even a reduction of 100% reduced the value of λ to < 1.0 (Fig.

6B). However, a significant reduction of λ to < 1.0 is possible when two or three of these vital rates (G_2, G_3 and P_5) are perturbed at a same time (Table 5).

Biological control as a tool for CBB management

Several methods, including biological, cultural and chemical control, have been tested and recommended to reduce CBB populations [3, 7, 32]. Biological control organisms have been recommended, including parasitoid wasps [33-35], entomopathogenic fungi (*B. bassiana* and *Metharizium anisopliae* (Metchnikoff) Sorokin) [36-39], nematodes [40, 41], and ants [42-44].

Ants and parasitoid wasps could help to reduce probabilities G_2 and G_3 or survival of larvae and pupae, respectively. Some studies have suggested that several species of ants can remove immature stages of the CBB from coffee fruits [42, 45, 46], and in many cases they make nests inside the fruits [46]. However, few studies have tested the potential of ants as predators of immature stages under laboratory or field conditions.

Varón *et al.* [45] demonstrated that under laboratory conditions five species of ants are potential predators of larvae and pupae of CBB: *Solenopsis geminata* (Fabricius), *Pheidole radoszkowskii* (Mayr), *Crematogaster crinosa* (Mayr), *Crematogaster curvispinosa* (Mayr) and *Crematogaster torosa* (Mayr) were able to predate 20 to 100% of larvae exposed inside acrylic boxes. However, in the field only *P. radoszkowskii* and *C. torosa* were able to remove larvae from fruits, and at lower

percentages (19 and 1%, respectively), and only *C. torosa* was able to remove pupae. The authors suggested that these lower percentages of predation in the field are a consequence of difficulty of entering coffee fruits through the hole made by the CBB; workers of *S. geminata* are too large.

Two small species of ants, *Wasmannia auropunctata* (Roger) and *Solenopsis picea* (Emery), were tested for potential to predate immature CBB stages in the lab, and their capacity to enter to the coffee fruits through holes made by the CBB [47]. These species removed 80% and 47%, respectively, of a mix of larvae and pupae from metal platforms placed over ant nests. However, only *W. auropunctata* was able to penetrate and remain inside the infested fruits. These two studies suggest that only *W. auropunctata, P. radoszkowskii* and *C. torosa* could be used in the field for biological control of larvae and pupae of the CBB.

W. auropunctata, or the little fire ant, is very common on coffee farms in Latin America, and has great capacity to predate adult CBB females [43]. In Puerto Rico, this species is named *albayalde* or *abayarde* and is commonly found in coffee farms in both sun and shade [44, 48, 49]. *W. auropunctata* significantly reduced CBB survival and damage to coffee, due its capacity to predate CBB adult females and prevent the colonization of coffee fruits [44, 50]. However, *W. auropunctata* is a problem for coffee production because its presence interferes with picking labor; farm workers avoid areas with high abundance of this species due to its painful bite [51].

In Puerto Rico there are no reports of *P. radoszkowskii* and *C. torosa,* but other species belonging to these genera have been reported on coffee farms. Smith [48] reported one species of *Crematogaster* (*C. steinheili* (Forel)), and two species of *Pheidole: P. fallax* (Mayr) and *P. subarmata* (Mayr). Newson [44] observed another three species of *Pheidole* into some coffee farms: *P. exigua* (Mayr)*, P. megacephala* (Fabricius) and *P. moerens* (Wheeler). This last was the most abundant; however, its presence in the coffee farms did not reduce the damage and survival of the CBB. Of these six species, only *P. megacephala* has been reported as an important predator of the CBB in Cuba [46].

Four species of parasitoid wasps have been identified as natural enemies of the CBB: *Cephalonomia stephanoderis* (Betrem)*, Prorops nasuta* (Waterson), *Phymastichus coffea* (La Salle) and *Heterospilus coffeicola* (Schmiedknecht). Of these, *C. stephanoderis, P. nasuta* and *H. coffeicola* have the capacity to predate immature stages of the CBB, mainly small larvae and eggs. The first two species also can parasite bigger larvae (instar II), prepupae and pupae [33, 34, 52]. In Puerto Rico, *C. stephanoderis* was observed occurring naturally into some coffee farms [53, 54].

Parasitoids, ants and predators can also reduce the survival of CBB adult females (P_5). All four species of parasitoids described above have the capacity to predate or parasite CBB females. For instance, *P. nasuta* and *C. stephanoderis* can penetrate the coffee fruit and complete their life cycles inside the fruit; once inside the fruit the female wasp paralyzes or kills the CBB female and uses its body as a barrier to

limit the entry of other organisms [52]. Feeding and oviposition of parasitoid wasps take place after CBB females or bigger larvae and pupae are paralyzed [33, 34, 52].

Several species of ants are known to predate adult CBBs: *W. auropunctata, S. picea, S. invicta* (Buren) and *Tetramollium simillium* (Roger) [43, 50, 55]. Another important predator of adult CBBs is the flat bark beetle *Cathartus quadricollis* (Guérin-Méneville); this insect has been found inside infested coffee fruits in Puerto Rico and Hawaii [53, 56]. Laboratory assays demonstrated the capacity of adults and larvae of this species to feed on all stages of CBB [56, 57].

More detailed and extensive studies on the diversity of ant species, parasitoids, and other predators of the CBB in Puerto Rico and other coffee-growing areas are needed. It is important to elucidate the full potential of these organisms as biocontrol of CBB with a focus on abundant and promising species of ants like *W. auropunctata* and *S. invicta,* which were able to reduce CBB survival and damage to coffee [44]. However, these predators of CBBs also play other roles in the complex web of trophic interactions on a coffee farms [58], so manipulating them may have unintended consequences.

Strategies for management of the CBB have focused on reducing the survival of adult females. Biological and cultural controls are the main tools for IPM programs for CBB. Chemical control using insecticides is limited in many coffee producing countries due to health and environmental concerns. Additionally, the frequent use of an insecticide leads to development of resistance; for instance, resistance of the CBB to

endosulfan has been reported, and endosulfan is one of the most used insecticides against the CBB [59].

The fungus *B. bassiana* is an important tool for biological control of the CBB [11, 12, 37, 38]. In Puerto Rico and other countries *B. bassiana* is commonly applied as Mycotrol® or Botanigard®. However, in this study mortality caused by *B. bassiana* was not common.

Other pathogenic fungi or bacteria that cause internal rots could be an additional factor for CBB mortality, or stimulate its migration to other fruits [6, 17]. Likewise, we observed more internal rot in fruits in which the CBBs were found dead or missing compared with those fruits in which CBBs were alive. It is likely that some of the rot pathogens or associated microorganisms killed the CBBs or induced them to abandon the fruits.

Four species of *Colletotrichum* (*C. fructicola, C. siamense, C. theobromicola* and *C. tropicale*) were recently reported as pathogens of coffee fruits, with the ability to cause internal and external rots; *Fusarium* was also isolated from internal rots [10]. Several species of *Fusarium* have been isolated from the body or gut of CBB [60-63]. Some species of *Fusarium* are weak or strong pathogens of insects; their mode of action can include penetration of the integument, release of toxins, or ectoparasitism [64]. For instance, *F. solani* is a weak pathogen of larva of the large elm bark beetle *Scolytus scolytus* [65]. However, organisms that can cause coffee fruit rots are not good candidates for control of the CBB.

Cultural practices mainly involve sanitation, that is, the periodic collection of ripe and raisin fruits during and after the harvest [32]. There are two important methods of sanitation: the first consists in the continuous collection of all ripe fruits left by the pickers during and after the harvest, on the plant and on the ground. This method is called in South America “re-re” (short for ‘recolección y repase’ or ‘collect and repeat’) [3, 66]. The second consists in the complete collection of all remaining fruits (green, ripe and raisin) after the main harvest; this method is known as ‘strip-picking’ [32].

‘Re-Re’ is commonly implemented where there are two coffee crops per year, as in Colombia and Brazil. In Hawaii and Puerto Rico, where there is only a single coffee crop per year, ‘strip-picking’ is more recommended [32]. In Puerto Rico, remaining fruits on the plant are the most important reservoirs for CBBs: one unharvested fruit can harbor up to 45 CBBs [67].

~~**Life table parameters**~~

Significant differences were observed in values of demographic parameters between populations reared in the lab vs. populations in the field. Overall, populations growing under lab conditions with constant abiotic variables (temperature and RH), few disturbances and an artificial diet with high nutritional content performed better (Table 3). Retrospective (LTREs) analyses showed that reduction in G_2 (transition from larva to pupa) and G_3 (transition from pupa to juvenile) probabilities made the largest contribution to the reduction in λ observed for populations in the field (Fig. 5 and Table

3). Prospective analysis also identified these two transitions as important targets for CBB management; these vital rates had higher values of elasticity.

CBB females expulse immature stages from the fruit under some circumstances, and they also conduct a rigorous hygiene of dead immature stages, which are removed rapidly from the fruit [17]. This report agrees with our results: we observed a marked decrease in the number of pupae compared with number of larvae counted. For instance, 297 larvae in instar I resulted in 147 pupae, suggesting that 51% of larvae died, were predated by ants and/or were expulsed from the fruit by adult CBBs before they became pupae. A more drastic loss was observed for G_3: 177 pupae resulted in only 24 juveniles; this means that 84% of pupae disappeared from the fruits. However, these decreases in G_2 and G_3 probabilities under natural conditions were not sufficient to reduce the value of λ to <1.0.

Relatively few studies have determined demographic parameters of CBB populations under field conditions. Baker *et al.* [17] and Ruiz and Baker [20] conducted experiments on coffee farms in Mexico and Colombia, respectively. Values reported for a coffee plantation in Mexico, with a mean temperature of 26°C were: λ = 1.067, r_m = 0.065, T = 44.7 days and DT = 10.7 days. In Colombia for two coffee plantations with a mean temperature of 20.7 to 21.6°C, values estimated were: λ = 1.060 to 1.070, r_m = 0.066, T = 48.5 to 54.3 days and DT = 10.5 to 10.7 days. Values of r_m, T and DT were similar to those estimated in this study in Puerto Rico with a mean temperature 21.8°C (r_m = 0.053, T=40.1 and DT = 13.1 days, Table 3). However, we observed a difference

in the value of λ, which was higher for our populations (1.32); this value corresponds to artificial infestations in field. To perform these infestations, we used CBBs reared in the lab on the artificial diet, which had the best performance, as mentioned above. One possible explanation could be related to the fecundity of CBB females, which was significantly higher for females reared in the lab vs. those that infested naturally (Fig. 3). Explanations for this difference in fecundity are discussed in more detail in the next section.

The first adult offspring in the field were observed 56 days after infestation, which suggests that developmental time from egg to adult was approximately 49 days. These results contrast with previous field observations made at similar mean temperatures. In a shade plot in Mexico with a mean temperature of 20.8°C and planted with *C. canephora* cv. Robusta, the first adults appeared between 42 to 49 days after infestation [17]. In Hawaii, in a plot planted with *C. arabica* cv. Typica exposed to full sun, with mean temperature of 20.6 °C (a mean temperature like those reported by Baker et al., [17]) the first adults appeared 47 days after infestation; while at 22.0°C (similar to that reported in this study), first adults appeared 43 days after infestation [68].

Hamilton *et al.* [68] suggested that fluctuations in daily temperatures can also influence developmental time: sites with higher daily ranges in temperatures had slower CBB development. For instance, in a site with a daily range of temperature of 14.9°C the first adults appeared at 51 days after infestation. Our results support these observations: the daily range in temperature was 14.1°C in this study, approximately 0.8

°C less than that reported by Hamilton *et al*. [68], and development time was longer (the first adults appeared 56 days after infestation). These results suggest that mean temperature is not the only factor that influences development time; there are other factors such as daily range in temperature, coffee species and varieties.

~~Comparisons between CBBs reared in the lab vs. CBBs growing on the field~~

Artificial infestations by CBBs reared in the lab were successful: 468 females infested and reproduced inside coffee fruits. After infesting the fruits, behavior of these CBBs was like that of CBBs that infested naturally. CBBs reared in the lab and those from the field were affected equally by abiotic (temperature and relative humidity) and biotic variables (ants, parasitoids, entomopathogenic fungi); no differences were observed in death and abandonment rate, and proportion of CBBs in positions A and B (Fig. 1 and 2). However, more CBBs from artificial infestation were in position D. In other words, CBBs reared in the lab were more likely to reach the endosperm and start to reproduce there (Fig. 2).

This high proportion of colonizing females in position D could be related to the frequency of males. When CBBs were reared in the artificial diet Cenibroca male offspring were found in all vials evaluated, and the number of males per vial increased through generations from 2.4 ± 0.3 (F_1) to 11.3 ± 0.3 (F_{10}) [21]. Higher frequency of males in the artificial diet could increase the possibility that more females are fertilized, reach the endosperm, and oviposit. In the field, Mariño *et al.* [69] evaluated CBB

reproduction and found that 61% of fruits did not contain males (this percentage only include fruits with juveniles and adults, which are the stages that can be sexed).

CBBs females reared in the lab and used to perform artificial infestations were more fertile, they produced almost 4x more offspring per fruit that CBBs from natural infestations (Fig. 3). This high fecundity could be associated with the food source and quality, and the temperature during the pre-oviposition period to which these females were exposed.

Food source, availability and quality have a direct impact on the growth, development, and reproduction of insects. In the case of herbivorous insects like the CBB, successful development and reproduction depends on the nutrients offered by its host plant. One of the most important elements that determines host quality is nitrogen [70, 71]. Several studies have demonstrated that the amount of nitrogen available during the development of larvae, pupae and adults has important effects on the future fecundity and fertility [71]. For example, females of the tortoise beetle *Paropsis atomaria* (Oliver) that feed on foliage of *Eucalyptus blakelyi* (Maiden) with high levels of nitrogen (4.12%) produced 5x more eggs than females that feed of foliage with low levels of nitrogen (1.15%) [72].

The artificial diets used to grow insects are usually a mixture of nutrients (carbohydrates, lipids and proteins), vitamins, salts and preservatives [73]. The artificial diet Cenibroca that we used to grow CBBs contains casein and yeasts [22]. Casein and

yeasts are important sources of nitrogen. The N content of casein is 14.2% [74] , and is almost 10x higher than N content of endosperm or almond from coffee fruits, which is 1.89% [75].

Finally, these higher proportions of females in position D from females reared on the artificial diets could also be related to temperature during the pre-oviposition period to which these females were exposed. CBBs reared on artificial diet and used to perform artificial infestations were maintained at a constant temperature of 25°C, while females from natural infestation were exposed to variables temperatures from 12°C to 34.1°C, with a daily range 14.1°C (Table 1). Jaramillo *et al.* [19] evaluated the effect of temperature on fecundity of CBB females reared in the lab on fruits of *C. arabica* cv. Ruiri at eight temperatures ranging from 15°C to 35°C. Females exposed to cold temperatures (15°C) were able to reach the endosperm but did not oviposit, while females exposed to warm temperatures (35°C) did not reach the endosperm. Bergamin [76] also suggested that exposition to low temperatures had a negative effect on the oviposition activity of CBB females.

Conclusions and Recommendations

This study is the first to report artificial infestations of CBBs in the field to perform elasticity and prospective perturbation analyses. These analyses identified which vital rates or CBB developmental stages are the best targets to improve CBB management programs. Results from elasticity analyses determined that survival of larvae, pupae and adult females are the three vital rates that most influenced population growth (λ).

Perturbation analyses showed that a successful Integrated Pest Management (IPM) program for the CBB should not be focused only in one stage or vital rate. When one vital rate including the three with highest sensitivity were perturbed at a time and the others remained constant, very high percentages of perturbation were necessary to reduce the value of λ to <1.0. IPM programs should be directed to enhance the diversity of natural enemies, mainly ants, parasitoids and predators, which have the capacity to affect or impact the survival of these three important targets. To increase the diversity of natural enemies of the CBB, the first steps could be the re-introduction of shade trees on sun coffee plantations and the reduction or elimination of chemical pesticides used to management the CBB and other coffee pests like the coffee leaf miner (*Leucoptera coffeela* Guérin-Méneville), which also reduce populations of natural enemies of the CBB [58].

Future investigation and experimentation are needed to determine the diversity, abundance and effectiveness of natural enemies (ants, parasitoids and other predators of the CBB) in Puerto Rico and other coffee-growing areas. It is important to focus on the role of these organisms as predators of larvae, pupae and CBB adult females.

Acknowledgments

We thank Rocío Rivera for help in the lab. This project was supported by USDA ARS Specific Cooperative Agreements 58-1245-4-082 and 58-1245-4-083 and by an Advanced Research Grant from the Puerto Rico Science and Technology Research Trust.

References

1. Vandermeer JH. Tropical agroecosystems. Boca Raton, Florida, USA: CRC press; 2002. 38 p.

2. Rizzuto MLQ, Rosales M. El mercado mundial del café: tendencias recientes, estructura y estrategias de competitividad. Visión Gerencial. 2014;(2):291-307.

3. Jaramillo J, Borgemeister C, Baker P. Coffee berry borer *Hypothenemus hampei* (Coleoptera: Curculionidae): searching for sustainable control strategies. Bull Entomol Res. 2006;96(3):223-33.

4. Rice RA. A place unbecoming: the coffee farm of northern Latin America. Geogr Rev. 1999;89(4):554-79.

5. FAO (Food and Agriculture Organization of the United Nations). Coffee Pocektbook 2015. Rome, Italy2015.

6. Damon A. A review of the biology and control of the coffee berry borer, *Hypothenemus hampei* (Coleoptera: Scolytidae). Bull Entomol Res. 2000;90(6):453-65.

7. Vega FE, Infante F, Castillo A, Jaramillo J. The coffee berry borer, *Hypothenemus hampei* (Ferrari)(Coleoptera: Curculionidae): a short review, with recent findings and future research directions. Terr Arthropod Rev. 2009;2:129-47.

8. Jaramillo J, Muchugu E, Vega FE, Davis A, Borgemeister C, Chabi-Olaye A. Some like it hot: the influence and implications of climate change on coffee berry borer (*Hypothenemus hampei*) and coffee production in East Africa. PLOS ONE. 2011;6(9):e24528.

9. Vega FE, Infante F, Johnson AJ. The genus *Hypothenemus*, with emphasis on *H. hampei*, the coffee berry borer. In: Vega FE, Hofstetter RW, editors. Bark Beetles: Biology and Ecology of Native and Invasive Species. 1st Ed ed. London, UK: Elsevier; 2015. p. 427-94.

10. Serrato-Diaz LM, Mariño YA, Bayman P. Pathogens Causing Anthracnose and Fruit Rots of Coffee Associated with the Coffee Berry Borer and the Entomopathogenic Fungus *Beauveria bassiana* in Puerto Rico. Phytopathology. 2020;110:1541-52.

11. de Souza RA, Pratissoli D, de Araujo Junior LM, de Assis Pinheiro J, Ataíde JO, Tamashiro LAG, et al. Mortality of Coffee Berry Borer, *Hypothenemus hampei* in Field, with Pre and Post Application of Entomopathogenic Fungus *Beauveria bassiana* (Balsamo) Associated to Emulsifiers. Int J Adv Res Sci Eng. 2020;7(5).

12. Hollingsworth RG, Aristizábal LF, Shriner S, Mascarin GM, Moral RdA, Arthurs SP. Incorporating *Beauveria bassiana* into an integrated pest management plan for Coffee Berry Borer in Hawaii. Front Sustain Food Syst. 2020;4:22.

13. Caswell H. Prospective and retrospective perturbation analyses: their roles in conservation biology. Ecology. 2000;81(3):619-27.

14. Caswell H. Sensitivity Analysis: Matrix Methods in Demography and Ecology. In: Myrskylä M, editor. Cham, Switzerland: Springer; 2019. p. 298.

15. Caswell H, Shyu E. Sensitivity analysis of periodic matrix population models. Theor Popul Biol. 2012;82(4):329-39.

16. Caswell H, Gassen NS. The sensitivity analysis of population projections. Demogr Res. 2015;33:801-40.

17. Baker P, Barrera J, Rivas A. Life-history studies of the coffee berry borer (*Hypothenemus hampei*, Scolytidae) on coffee trees in southern Mexico. J Appl Ecol. 1992:656-62.

18. Romero J, Cortina H. Tablas de vida de *Hypothenemus hampei* (Coleoptera: Curculionidae: Scolytinae) sobre tres introducciones de café. Rev Colomb Entomol. 2007;33(1):10-6.

19. Jaramillo J, Chabi-Olaye A, Kamonjo C, Jaramillo A, Vega F, Poehling HM, et al. Thermal tolerance of the coffee berry borer *Hypothenemus hampei*: Predictions of climate change impact on a tropical insect pest. PLOS ONE. 2009;4(8):e6487.

20. Ruiz R, Baker P. Life table of *Hypothenemus hampei* (Ferrari) in relation to coffee berry phenology under Colombian field conditions. Sci Agr. 2010;67(6):658-68.

21. Mariño YA, Verle Rodrigues JC, Bayman P. *Wolbachia* affects reproduction and population dynamics of the coffee berry borer (*Hypothenemus hampei*): Implications for biological control. Insects. 2017;8(1):8.

22. Portilla M. Mass rearing technique for *Cephalonomia stephanoderis* (Hymenoptera: Bethylidae) on *Hypothenemus hampei* (Coleoptera: Scolytidae) developed using Cenibroca artificial diet. Rev Colomb Entomol. 1999;25:57-66.

23. Gallardo F, González O. Manejo integrado de la broca del café en Puerto Rico. San Juan, Puerto Rico: Departamento de Agricultura de Puerto Rico, 2015.

24. Bustillo A, Cárdenas R, Villalba D, Benavides P, Orozco J, Posada F. Manejo integrado de la broca del café *Hypothenemus hampei* (Ferrari) en Colombia. Cenicafé, Chinchiná, Colombia. 1998;134.

25. Leslie PH. On the use of matrices in certain population mathematics. Biometrika. 1945;33(3):183-212.

26. Caswell H. Matrix population models: construction, analysis, and interpretation. Second edition ed. Sunderland, Massachusetts, USA: Sinauer Associates; 2001.

27. Carslake D, Stuart T, Hodgson D. Patterns and rules for sensitivity and elasticity in population projection matrices. Ecology. 2009;90(11):3258-67.

28. Caswell H. A general formula for the sensitivity of population growth rate to changes in life history parameters. Theor Popul Biol. 1978;14(2):215-30.

29. Stubben C, Milligan B. Estimating and analyzing demographic models using the popbio package in R. J Stat Softw. 2007;22(11):1-23.

30. R Core Development Team. R: a languaje and environment for statistical computing. Version 3.4.3 2017. Available from: http://www.R.project.org/.

31. Benton TG, Grant A. Elasticity analysis as an important tool in evolutionary and population ecology. Trends Ecol Evol. 1999;14(12):467-71.

32. Aristizábal LF, Johnson M, Shriner S, Hollingsworth R, Manoukis NC, Myers R, et al. Integrated pest management of coffee berry borer in Hawaii and Puerto Rico: current status and prospects. Insects. 2017;8(4):123.

33. Lauzière I, Brodeur J, Pérez-Lachaud G. Host stage selection and suitability in *Cephalonomia stephanoderis* Betrem (Hymenoptera: Bethylidae), a parasitoid of the coffee berry borer. Biol Control. 2001;21(2):128-33.

34. Benavides P, Bustillo A, Portilla M, Orozco J, editors. Classical biological control of coffee berry borer, *Hypothenemus hampei* (Coleoptera: Scolytidae) in Colombia with African parasitoids. Proceedings, 1st International Symposium on Biological Control of Arthropods, Honolulu, Hawaii; 2003.

35. Jaramillo J, Bustillo A, Montoya E, Borgemeister C. Biological control of the coffee berry borer *Hypothenemus hampei* (Coleoptera: Curculionidae) by *Phymastichus coffea* (Hymenoptera: Eulophidae) in Colombia. Bull Entomol Res. 2005;95(5):467-72.

36. De la Rosa-Reyes W, Godinez-Aguilar J, Alatorre-Rosas R. Biological activity of five strains of *Metarhizium anisopliae*, upon the coffee berry borer *Hypothenemus hampei* (Col.:Scolytidae). Entomophaga. 1995;40(3-4):403-12.

37. De La Rosa W, Alatorre R, Barrera J, Toriello C. Effect of *Beauveria bassiana* and *Metarhizium anisopliae* (Deuteromycetes) upon the coffee berry borer (Coleoptera: Scolytidae) under field conditions. J Econ Entomol. 2000;93(5):1409-14.

38. Cruz LP, Gaitan AL, Gongora CE. Exploiting the genetic diversity of *Beauveria*

bassiana for improving the biological control of the coffee berry borer through the use of strain mixtures. Appl Microbiol Biotechnol. 2006;71(6):918-26.

39. Pava-Ripoll M, Posada FJ, Momen B, Wang C, Leger RS. Increased pathogenicity against coffee berry borer, *Hypothenemus hampei* (Coleoptera: Curculionidae) by *Metarhizium anisopliae* expressing the scorpion toxin (AaIT) gene. J Invertebr Pathol. 2008;99(2):220-6.

40. Allard G, Moore D. *Heterorhabditis* sp. nematodes as control agents for coffee berry borer, *Hypothenemus hampei* (Scolytidae). J Invertebr Pathol. 1989;54(1):45-8.

41. Castillo A, Infante F, Barrera JF, Carta L, Vega FE. First field report of a nematode (Tylenchida: Sphaerularioidea) attacking the coffee berry borer, *Hypothenemus hampei* (Ferrari)(Coleoptera: Scolytidae) in the Americas. J Invertebr Pathol. 2002;79:199-202.

42. Bustillo AE, Cardenas R, Posada FJ. Inimigos Naturais e Competidores da Broca-do-Café *Hypothenemus hampei* (Ferrari)(Coleoptera: Scolytidae) na Colômbia. Neotrop Entomol. 2002;31(4):635-9.

43. Armbrecht I, Gallego MC. Testing ant predation on the coffee berry borer in shaded and sun coffee plantations in Colombia. Entomol Exp Appl. 2007;124(3):261-7.

44. Newson J. Conservation biocontrol of the coffee berry borer in coffee farms through ant predation. Michigan, USA: University of Michigan, Ann Arbor; 2020.

45. Varón EH, Hanson P, Borbón O, Carballo M, Hilje Quirós L. Potencial de hormigas como depredadoras de la broca del café (*Hypothenemus hampei*) en Costa Rica. 2004.

46. Vázquez L, Jiménez EB, Claro OE, Brito YM, Simonetti JA. Observaciones sobre enemigos naturales de la broca del café (*Hypothenemus hampei* Ferrari) en Cuba. Fitosanidad. 2006;10(4):307-8.

47. Morris JR, Perfecto I. Testing the potential for ant predation of immature coffee berry borer (*Hypothenemus hampei*) life stages. Agric Ecosyst Environ. 2016;233:224-8.

48. Smith M. The relationship of ants and other organisms to certain scale insects on coffee in Puerto Rico. J Agr U Puerto Rico. 1942;26(2):21-39.

49. Perfecto I, Vandermeer J. Antagonism between *Anolis* spp. and *Wasmannia auropunctata* in coffee farms on Puerto Rico: Potential complications of biological control of the coffee berry borer. Caribb J Sci. 2020;50(1):43-7.

50. Gonthier DJ, Ennis KK, Philpott SM, Vandermeer J, Perfecto I. Ants defend coffee from berry borer colonization. BioControl. 2013;58(6):815-20.

51. Wetterer JK, Porter SD. The little fire ant, *Wasmannia auropunctata*: distribution, impact and control. Sociobiology. 2003;42:1-41.

52. Infante F, Mumford J, Baker P. Life history studies of *Prorops nasuta*, a parasitoid of the coffee berry borer. BioControl. 2005;50(2):259-70.

53. González OP. Dinámica poblacional de la broca del café *Hypothenemus hampei* (Ferrari) y sus enemigos naturales en el cultivo de café *Coffea arabica* L. en Puerto Rico. Mayagüez, Puerto Rico: University of Puerto Rico; 2013.

54. Gallardo-Covas F. Biological control of insect pests in Puerto Rico. J Agr U Puerto Rico. 2017:153-63.

55. Ropero G, Armbrecht I. Depredación por hormigas sobre la broca del café *Hypothenemus hampei* (Curculionidae: Scolytinae) en cafetales cultivados bajo dos niveles de sombra en Colombia. Manejo Integrado de Plagas y Agroecología. 2005;76:32-40.

56. Follett PA, Kawabata A, Nelson R, Asmus G, Burt J, Goschke K, et al. Predation by flat bark beetles (Coleoptera: Silvanidae and Laemophloeidae) on coffee berry borer (Coleoptera: Curculionidae) in Hawaii coffee. Biol Control. 2016;101:152-8.

57. Laiton LA, Constantino LM, Benavides P. Predatory capacity of *Cathartus quadricollis* and *Ahasverus advena* (Coleoptera: Silvanidae) on *Hypothenemus hampei* (Coleoptera: Curculionidae) under laboratory conditions. Rev Colomb Entomol. 2018;44(2):200-5.

58. Perfecto I, Vandermeer J. Coffee agroecology: a new approach to understanding agricultural biodiversity, ecosystem services and sustainable development. New York, USA: Routledge; 2015.

59. Brun L-O, Marcillaud C, Gaudichon V, Suckling D. Cross resistance between insecticides in coffee berry borer, *Hypothenemus hampei* (Coleoptera: Scolytidae) from New Caledonia. Bull Entomol Res. 1994;84(2):175-8.

60. Kumar P, Prakasan C, Vijayalakshmi C. Record of entomopathogenic fungi on *Hypothenemus hampei* (Ferrari) from South India. J Coffee Res. 1994;24(2):119-20.

61. Balakrishnan M, Vijayan V, Sreedharan K, Bath P. New fungal associates of the coffee berry borer *Hypothenemus hampei*. J Coffee Res. 1995;25:52-4.

62. Rojas MG, Morales-Ramos JA, Harrington TC. Association between *Hypothenemus hampei* (Coleoptera: Scolytidae) and *Fusarium solani* (Moniliales: Tuberculariaceae). Ann Entomol Soc Am. 1999;92(1):98-100.

63. Carrión G, Bonet A. Mycobiota associated with the coffee berry borer

(Coleoptera: Scolytidae) and its galleries in fruit. Ann Entomol Soc Am. 2004;97(3):492-9.

64. Teetor GH, Roberts DW. Entomogenous *Fusarium* species. Mycopathologia. 1983;84(1):3-16.

65. Barson G. *Fusarium solani*, a weak pathogen of the larval stages of the large elm bark beetle Scolytus scolytus (Coleoptera: Scolytidae). J Invertebr Pathol. 1976;27(3):307-9.

66. Benavides P. El repase: Actividad para el manejo de la broca del café en Colombia. Cenicafé, Chinchiná, Colombia. 2012;46:1.

67. Mariño YA, Vega VJ, García JM, Verle Rodrigues JC, García NM, Bayman P. The coffee berry borer (Coleoptera: Curculionidae) in Puerto Rico: Distribution, infestation, and population per fruit. J Insect Sci. 2017;17(2).

68. Hamilton LJ, Hollingsworth RG, Sabado-Halpern M, Manoukis NC, Follett PA, Johnson MA. Coffee berry borer (*Hypothenemus hampei*)(Coleoptera: Curculionidae) development across an elevational gradient on Hawai ‘i Island: Applying laboratory degree-day predictions to natural field populations. PLOS ONE. 2019;14(7):e0218321.

69. Mariño YA, Pérez M-E, Gallardo F, Trifilio M, Cruz M, Bayman P. Sun vs. shade affects infestation, total population and sex ratio of the coffee berry borer (*Hypothenemus hampei*) in Puerto Rico. Agric Ecosyst Environ. 2016;222:258-66.

70. Awmack CS, Leather SR. Host plant quality and fecundity in herbivorous insects. Annu Rev Entomol. 2002;47(1):817-44.

71. Leather SR. Factors affecting fecundity, fertility, oviposition, and larviposition in insects. Insect reproduction: CRC Press; 1995. p. 143-74.

72. Ohmart C, Stewart L, Thomas J. Effects of nitrogen concentrations of *Eucalyptus blakelyi* foliage on the fecundity of *Paropsis atomaria* (Coleoptera: Chrysomelidae). Oecologia. 1985;68(1):41-4.

73. Lapointe SL, Evens TJ, Niedz RP. Insect diets as mixtures: optimization for a polyphagous weevil. J Insect Physiol. 2008;54(7):1157-67.

74. Purevsuren B, Davaajav Y. Investigation on pyrolysis of casein. J Therm Anal Calorim. 2001;66(3):743-8.

75. Sadeghian S, Mejía B, Arcila J. Composición elemental de frutos de café y extracción de nutrientes por la cosecha en la zona cafetera de Colombia. Cenicafé, Chinchiná, Colombia. 2007;57(4):251-61.

76. Bergamin J. Contribution to the biology knowledge of coffee berry borer *Hypothenemus hampei* (Ferr)(Coleoptera: Ipidae). Arquivos do Instituto Biológico. 1943;14:31-72.

Figure 1

Click here to access/download;Figure;Fig 1. Proportion of alive, dead and missing CBBs.TIF

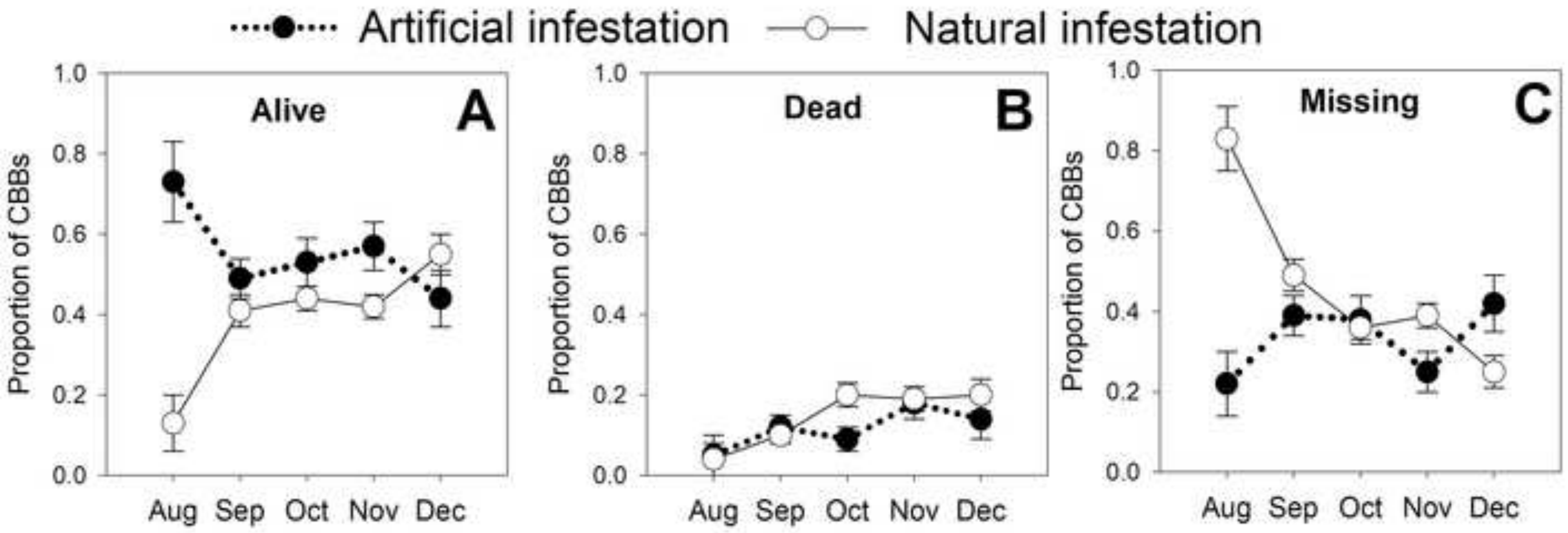

Figure 2

Click here to access/download;Figure;Fig 2. Progress of CBBs in penetrating fruits.TIF

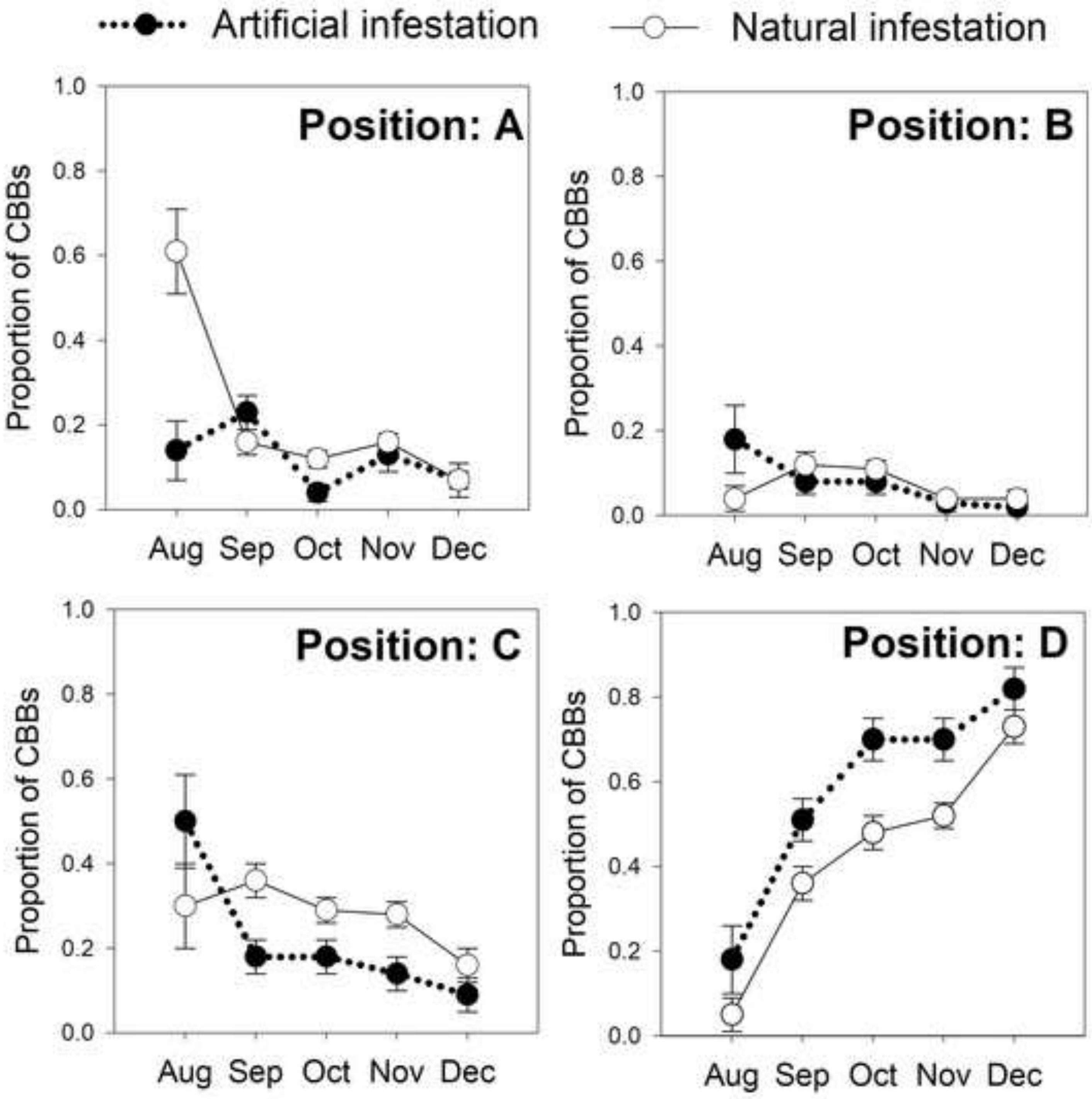

Click here to access/download;Figure;Fig 3. Monthly changes in total number of CBBs per fruit.TIF

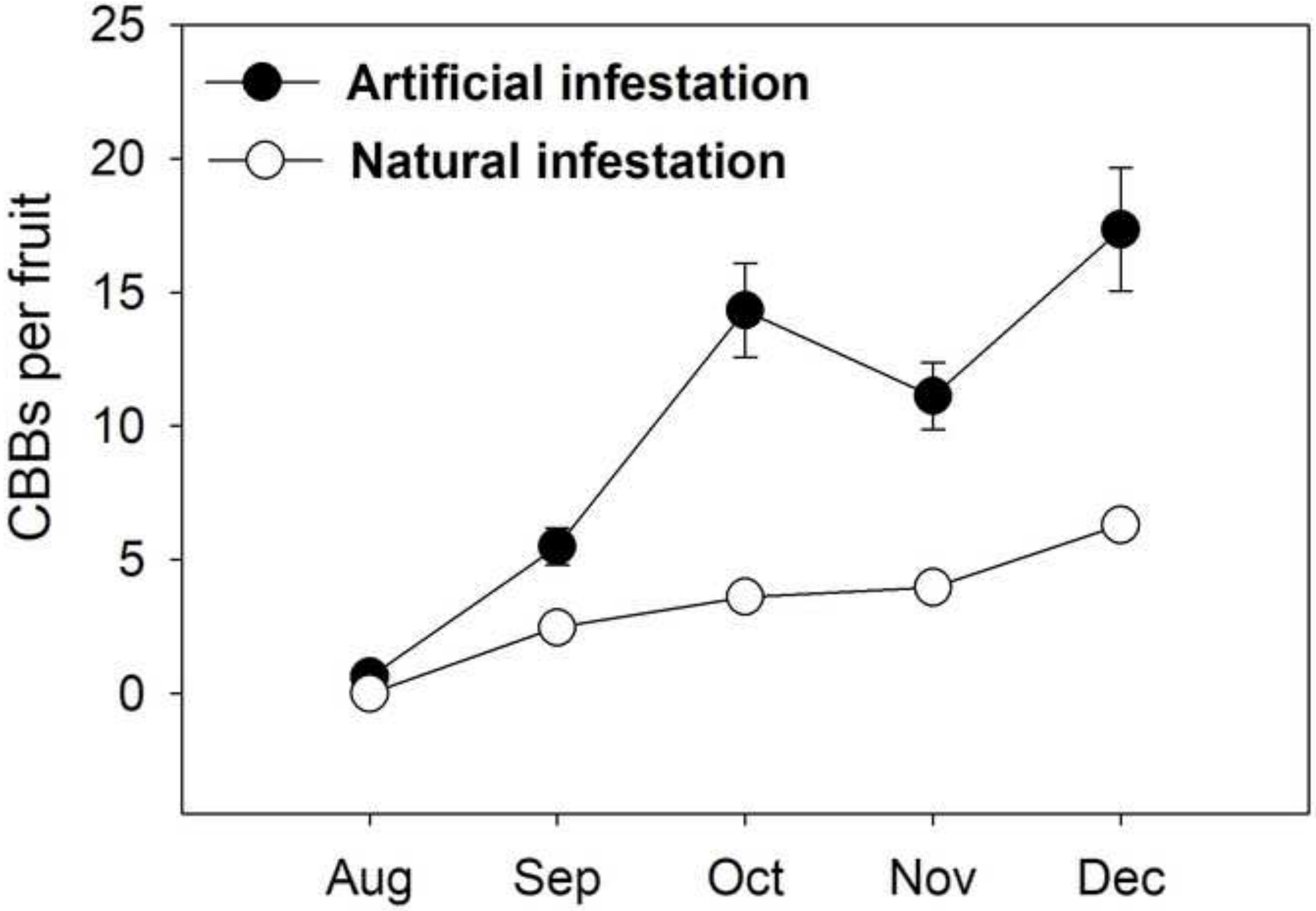

Figure 4

Click here to access/download;Figure;Fig 4. Number of CBBs per fruit in artificial infestation.TIF

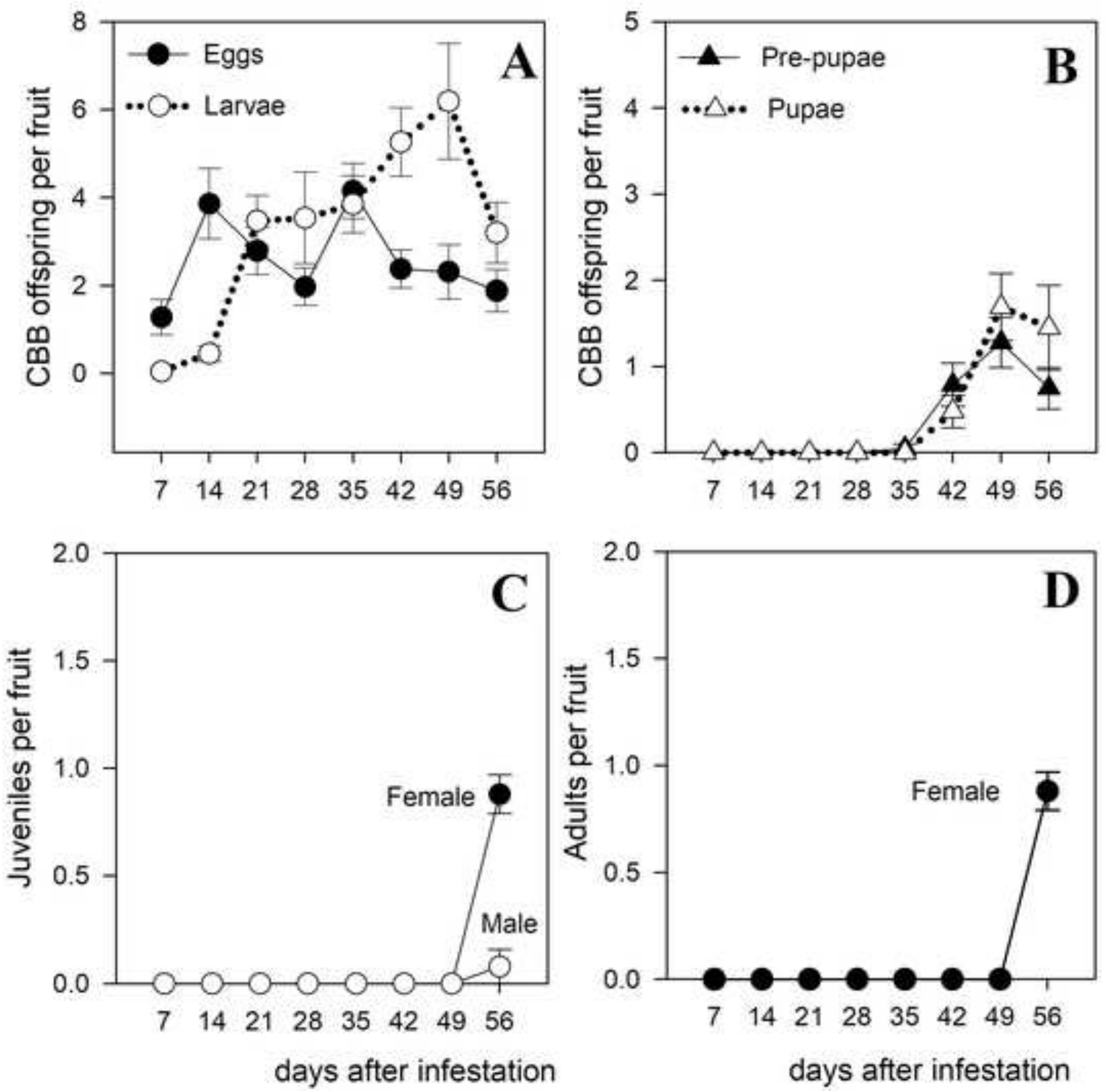

Figure 5

Click here to access/download;Figure;Fig 5. Life table response experiment (LTRE) for CBB.TIF

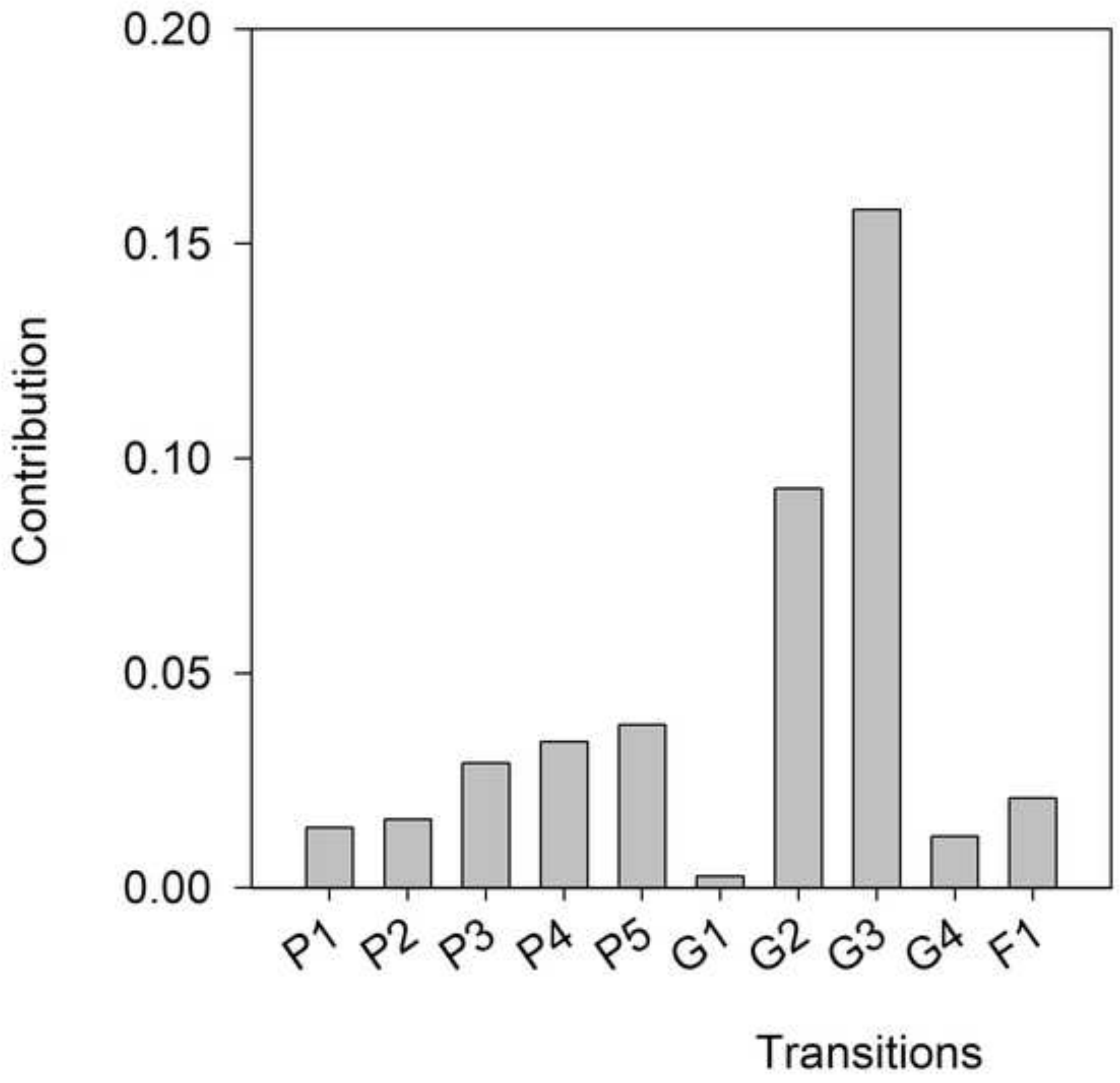

Figure 6

Click here to access/download;Figure;Fig 6. Perturbation analysis for the CBB one stage.TIF

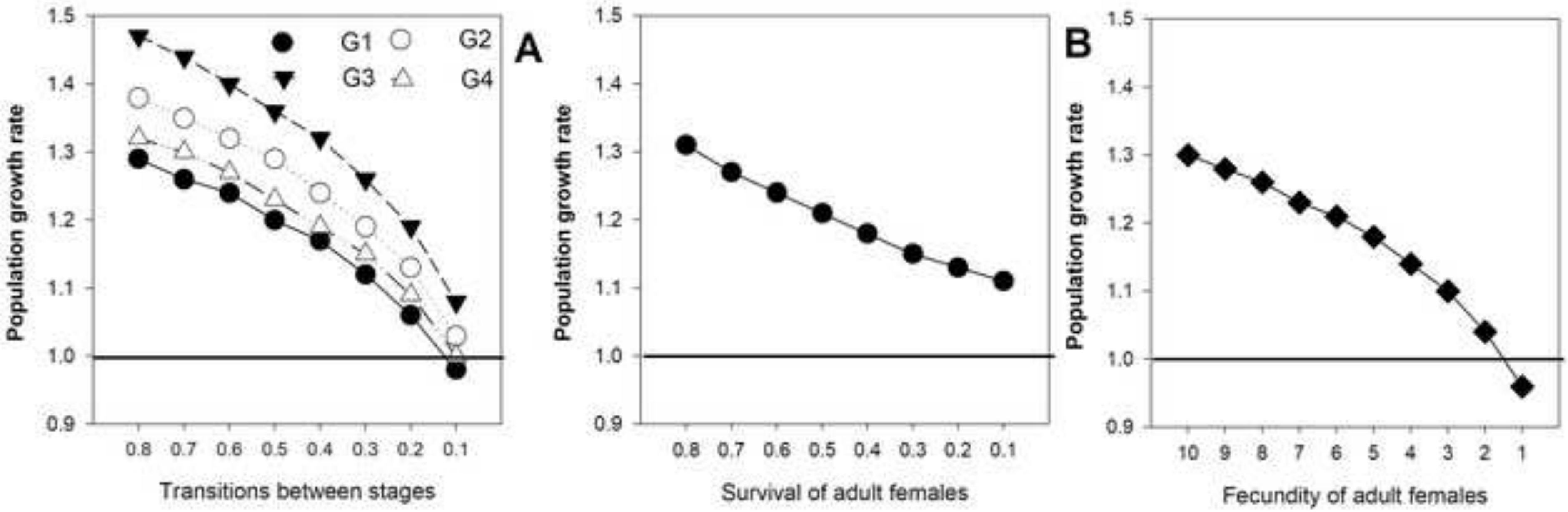

Figure 7

Click here to access/download;Figure;Fig 7. Perturbation analysis for the CBB two stages.TIF

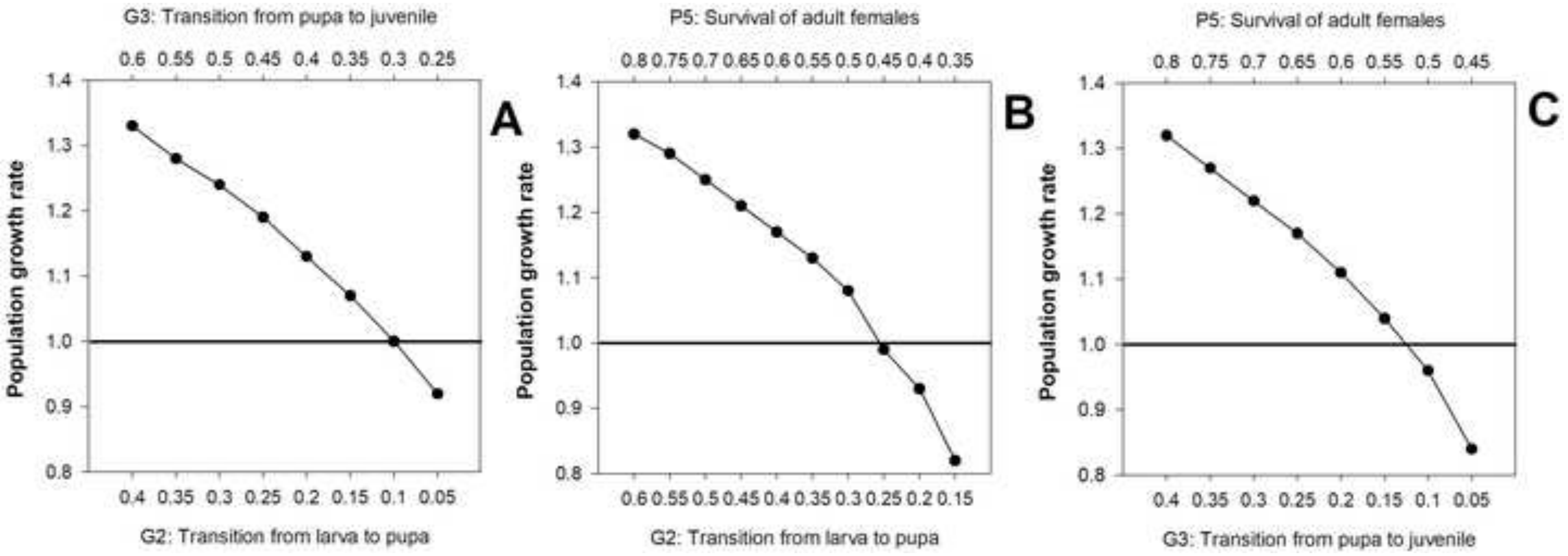

Click here to access/download
Supporting Information
renamed_558ad.docx
