# Supplementary material for: Demography and perturbation analyses of the coffee berry borer Hypothenemus hampei (Coleoptera: Curculionidae): Implications for management
Source: PLoS One. 2021 Dec 14;16(12):e0260499. doi: 10.1371/journal.pone.0260499 (PMC8670699; doi:10.1371/journal.pone.0260499)
Supplement: Attachment — Submitted filename: Marino_PLOSreview_ok.docx [file pone.0260499.s003.docx]

The study by Marino et al. describes CBB development times under natural and laboratory conditions and conducts perturbation and elasticity analyses to determine which stages in the life cycle contribute most to population growth rate. The study is well-thought out, nicely written, the analyses seem appropriate, and the discussion is comprehensive. Overall, this represents an important contribution to the literature on this worldwide pest of coffee. I have some minor comments which should easily be addressed. I enjoyed reading this manuscript and look forward to seeing it published.

L87-88: How did you decide on the environmental conditions to maintain the lab colony (why 25 C and 80-96% RH)?

L124-125: Were entomological sleeves placed around the entire branch? If not, how do you know that artificial infestations were the result of lab-reared CBB and not naturally occurring CBB from neighboring trees/branches?

L128: You might define larvae I vs. larvae II and pre-pupae vs. pupae.

L135-138: Please explain why mortality by Bb and internal fruit rot was evaluated and how these variables relate to the overall goals of the study.

L141: “Fully feral” is the same as natural, correct? I think best to stick to “natural field-infestation” so as not to confuse the reader (it could be inferred that these were infestations in wild coffee).

L1579-159: Are these daily temp and RH ranges, or ranges across the entire sampling period?

Table 1: Why is SE only shown for mean temp?

L172: Why were lab populations followed for 50 days and artificially infested populations followed for 56 days? Was this to account for the two days over which infestation could have occurred for artificial infestations?

L189: Here you say lab populations were evaluated at 56 days not 50 days?

L270-275: This should be moved to discussion.

L327: How could both juveniles and adults show up on same day (day 56)? Wouldn’t the presence of adults on day 56 mean that juveniles had to be present before that? Does this mean you might have missed the first appearance of juveniles?

Table 2: Change “media” to “mean”. Please include SE for min and max.

L523, L528: Change “parasite” to “parasitize”.

L526: Add “other” before predators.

L559-560: Yes, but this is because B. bassiana was not sprayed during your study, correct? Please include commonly reported rates of mortality due to B. bassiana from other studies.

L587: change to “more often recommended”.

L588: This is also true for other regions, such as Hawaii (see Johnson & Fortna et al. 2019).

L602: add “adult”

L648: Please explain why coffee species and variety would affect CBB development times.

L691: I am confused by the statement “endosperm or almond from coffee fruits”. Please revise.

L704: Exposition? Do you mean exposure?
