# Supplementary material for: Demography and perturbation analyses of the coffee berry borer Hypothenemus hampei (Coleoptera: Curculionidae): Implications for management
Source: PLoS One. 2021 Dec 14;16(12):e0260499. doi: 10.1371/journal.pone.0260499 (PMC8670699; doi:10.1371/journal.pone.0260499)
Supplement: Attachment — Submitted filename: Response to Reviewers.docx [file pone.0260499.s006.docx]

Dear reviewers:

Thanks a lot for reviewed our manuscript entitled “Demography and perturbation analyses of the coffee berry borer *Hypothenemus hampei* (Coleoptera: Curculionidae): implications for management”. We found your comments to be very useful and constructive, and we have incorporated most of them. The list of comments and our responses (in **bold**) follow below.

**Reviewer #2.**

L9: change to “entire life cycle occurs inside the fruit where it is well-protected.”

**We made the change, see L9.**

L10: change to “would shed light on the population dynamics of this pest…”

**We made the change, see L10 -11.**

L20: change comma to semi-colon

**We made the change, see L20.**

L21-22: change to “indicated that effective CBB management should consider multiple developmental stages;…”

**We made the change, see L22.**

L48: change to “which cause internal fruit rot”

**We made the change, see L48.**

L50: change to “because chemical and biological control measures are only effective

during the short period of time when adult females are out searching for new fruits to infest.”

**We made the change, see L50-51.**

L164-166: You need to include here that entomological sleeves were used to cover the experimental branches.

**We used plastic bags to cover the entire branch. We added a phrase to specify that plastic bags were used as entomological sleeves, see L 163-164.**

L177: note in parentheses at first mention that Bb refers to B. bassiana

**To be consistent we changed Bb to *B. bassiana*, as you recommended in the first revision, see L 181.**

L194-196: Move this up to start the paragraph at L177.

**We made the change, see L 177-179.**

L214: You need to include the information in the text here on why you used different evaluation periods for lab and field (50 vs. 56 days). Please include the information you put in your response to reviewers into the manuscript here.

**We added the information, see L 212 -216.**

L530: indicating that?? farmers should use commercial formulations to manage CBB instead of relying on naturally occurring B. bassiana strains? This thought seems incomplete as written.

**We added a sentence recommending the application of both commercial and local strains of *B. bassiana* to reduce the damage caused by the coffee berry borer, see L 532 – 533.**

L536: Why do you think the mortality by natural Bb was higher in the study reported by Wraight et al? Are the farms in PR considerably drier and hotter than farms in Hawaii where feral strains of Bb thrive?

**There are two possible explanations for the difference in the natural presence or mortality of the CBB caused by *B. bassiana* between Hawaii and Puerto Rico. The first, we agree with you, the differences in temperature and relative humidity could be affecting the presence and development of natural infections with Bb.**

**Farms from Puerto Rico experienced hotter and dryer days; for example, in our sampling site at 434 m.a.s.l. the temperatures ranged from 13 to 34°C and the minimum relative humidity was 47.6%; these measures are from September to November. While in Hawaii, for this same interval of time in a site located at a similar altitude 427 m.a.s.l, the temperatures ranged from 18.9 to 29.8°C and the minimum relative humidity was 66.7%.**

**Another reason could be that our sampling site was located at an experimental farm with similar management to a commercial Farm. Wraight et al, 2018 found that the natural presence of Bb was significantly less common in commercial farms than feral sites.**

**Environmental data from Hawaii were taken from Hamilton et al. 2019. Coffee berry borer (*Hypothenemus hampei*) (Coleoptera: Curculionidae) development across an elevational gradient on Hawai‘I Island: Applying laboratory degree-day predictions to natural field populations. PLOs ONE** 14(7): e0218321. https://

doi.org/10.1371/journal.pone.0218321.

**Wraight et al. 2018. Prevalence of naturally-occurring strains of *Beauveria bassiana* in populations of coffee berry borer *Hypothenemus hampei* on Hawai'i Island, with observations on coffee plant-H. hampei-B. bassiana interactions. *J. Invertebr. Pathol.* 156, 54–72. doi: 10.1016/j.jip.2018.07.008.**

L552, 555, 559: change sanitization to sanitation

**We made the change, see L 555, 558, 562.**

L590-603: Here you should include the information from your response on why you used 25 °C instead of 27 °C.

**We added the information, see L 606-608.**
